# Methane Inhibition Alters the Microbial Community, Hydrogen Flow, and Fermentation Response in the Rumen of Cattle

**DOI:** 10.3389/fmicb.2016.01122

**Published:** 2016-07-19

**Authors:** Gonzalo Martinez-Fernandez, Stuart E. Denman, Chunlei Yang, Jane Cheung, Makoto Mitsumori, Christopher S. McSweeney

**Affiliations:** ^1^CSIRO, Agriculture and Food, Queensland Bioscience PrecinctSt Lucia, QLD, Australia; ^2^Institute of Dairy Science, MoE Key Laboratory of Molecular Animal Nutrition, College of Animal Sciences, Zhejiang UniversityHangzhou, China; ^3^NARO Institute of Livestock and Grassland ScienceTsukuba, Japan

**Keywords:** rumen, microbial community, metabolites, methane, hydrogen, 16S sequencing

## Abstract

Management of metabolic hydrogen ([H]) in the rumen has been identified as an important consideration when reducing ruminant CH_4_ emissions. However, little is known about hydrogen flux and microbial rumen population responses to CH_4_ inhibition when animals are fed with slowly degradable diets. The effects of the anti-methanogenic compound, chloroform, on rumen fermentation, microbial ecology, and H_2_/CH_4_ production were investigated *in vivo*. Eight rumen fistulated Brahman steers were fed a roughage hay diet (Rhode grass hay) or roughage hay:concentrate diet (60:40) with increasing levels (low, mid, and high) of chloroform in a cyclodextrin matrix. The increasing levels of chloroform resulted in an increase in H_2_ expelled as CH_4_ production decreased with no effect on dry matter intakes. The amount of expelled H_2_ per mole of decreased methane, was lower for the hay diet suggesting a more efficient redirection of hydrogen into other microbial products compared with hay:concentrate diet. A shift in rumen fermentation toward propionate and branched-chain fatty acids was observed for both diets. Animals fed with the hay:concentrate diet had both higher formate concentration and H_2_ expelled than those fed only roughage hay. Metabolomic analyses revealed an increase in the concentration of amino acids, organic, and nucleic acids in the fluid phase for both diets when methanogenesis was inhibited. These changes in the rumen metabolism were accompanied by a shift in the microbiota with an increase in Bacteroidetes:Firmicutes ratio and a decrease in Archaea and Synergistetes for both diets. Within the Bacteroidetes family, some OTUs assigned to *Prevotella* were promoted under chloroform treatment. These bacteria may be partly responsible for the increase in amino acids and propionate in the rumen. No significant changes were observed for abundance of fibrolytic bacteria, protozoa, and fungi, which suggests that fiber degradation was not impaired. The observed 30% decrease in methanogenesis did not adversely affect rumen metabolism and the rumen microbiota was able to adapt and redirect [H] into other microbial end-products for both diets. However, it is also required dietary supplements or microbial treatments to capture the additional H_2_ expelled by the animal to further improve rumen digestive efficiency.

## Introduction

The principal greenhouse gas emitted from livestock is enteric CH_4_ which represents between 7 and 18% of total anthropogenic emissions (Hristov et al., 2013). Methane produced as an end product of fermentation constitutes an energy loss from digested feed (estimated between 2 and 12% of gross energy intake; Johnson and Johnson, 1995). If methanogenesis was inhibited and the available [H] was redirected into alternative energy-yielding metabolic pathways increased productivity could be expected. Thus, reducing CH_4_ production could potentially improve productivity for the same energetic intake by the animal provided rumen metabolism is not compromised.

The fermentation of feedstuff in the rumen by bacteria, protozoa, and fungi produces H_2_ which is used by some bacteria and methanogens to obtain energy while generating metabolic end products through this process. Methanogenic archaea are the main consumers of the H_2_ in this ecosystem, producing CH_4_ as the end product (Ungerfeld and Kohn, 2006; Janssen, 2010). It has been assumed that H_2_ accumulation resulting from the inhibition of methanogenesis will impair fiber digestion and fermentation (Wolin et al., 1997; McAllister and Newbold, 2008; Janssen, 2010). However, Mitsumori et al. (2012) found that inhibition of methanogenesis by bromochloromethane (BCM) in goats (around 80% CH_4_ decrease) dramatically increased H_2_ expelled without affecting dry matter intake (DMI) and feed digestibility. BCM has been used in numerous studies to decrease CH_4_ production in ruminants and it is considered one of the most effective inhibitors of methanogenesis (Denman et al., 2007; Goel et al., 2009; Abecia et al., 2012; Mitsumori et al., 2012). The restrictions on use of BCM due to its ozone depleting capacity have led to other halogenated methanogenesis inhibitors such as chloroform and bromoethanesulfonate (BES) being used in ruminant research as experimental models (Dong et al., 1999; Ungerfeld et al., 2004; Knight et al., 2011). Chloroform appears to decrease rumen CH_4_ production to the same extent as BCM with little or no adverse effect on rumen fermentation in dairy cows and *in vitro* (Knight et al., 2011; Hwang et al., 2012). Chloroform, like BCM, interferes with the transfer of the methyl group to methyl-coenzyme M (CoM) at the cobamide-dependent methyl transferase step of the methanogenesis pathway (Gunsalus and Wolfe, 1978; Graham and White, 2002).

Management of H_2_ in the rumen was identified as an important consideration when inhibiting ruminant CH_4_ emissions since it is assumed that accumulation of H_2_ would inhibit the re-oxidation of NADH and adversely affect fermentation (Wolin et al., 1997; Joblin, 1999). However, H_2_ is rarely measured while much effort is expended on quantifying the amounts of CH_4_ formed in animal studies. Recently three studies have measured both the expelled CH_4_ and H_2_ simultaneously in cattle and dairy cows. Rooke et al. (2014) showed significant differences on CH_4_ and H_2_ emissions between diets when cattle were fed a high-concentrate or a mixed forage-concentrate diet. Veneman et al. (2015) observed an increase in expelled H_2_ when methanogenesis was inhibited in dairy cows treated with nitrates or linseed oil and fed with a total mixed ration. Significant increases in expelled H_2_ occurred when methanogenesis was inhibited in dairy cows fed with a total mixed ration and treated with 3-nitrooxypropanol with non-detrimental effects on feed intake or apparent total tract digestibility (Hristov et al., 2015). In fact, a recent meta-analysis (Ungerfeld, 2015) of *in vitro* trials observed a greater accumulation of H_2_ when inhibiting methanogenesis in incubations with increasing concentrate substrate. However, little is known about rumen fermentation and microbial population responses to CH_4_ inhibition when animals are fed with slowly degradable diets, such as low quality roughage hay. With the exception of a study conducted by Vyas et al. (2016), there is scarce information about the effects of inhibiting methanogenesis with different types of diet in the same *in vivo* study.

The aims of the present study were to analyze the effect of chloroform on CH_4_ production, [H] flux, and subsequent responses in rumen fermentation and microbial community composition of cattle on diets of varying quality. Three dose levels of chloroform were administered into the rumen of steers fed *ad libitum* on two different diets of roughage:concentrate (60:40) or roughage hay. It was hypothesized that lesser amounts of H_2_ relative to intake would accumulate in the roughage fed animals compared to those receiving a roughage:concentrate diet due to a shift in fermentation to reductive processes that will consume more reducing equivalents, resulting in less energy lost to the animal.

## Materials and Methods

The experimental protocol complied with the Australian Code for the Care and Use of Animals for Scientific Purposes (eighth edition, 2013) and was approved by the local Animal Experimentation and Ethics Committee (A18/2013).

### Experimental Design and Sampling

Eight rumen-fistulated Brahman (*Bos indicus*) steers (mean, live weight LW, 288 ± 7 kg) were used in the experiment. Animals were randomly allocated to two groups (four animals per group), each group receiving a different *ad libitum* diet twice per day. One group was fed a roughage hay diet (Rhode grass hay; chemical composition: DM, 881 g/kg fresh matter; in g/kg of DM: OM, 802; CP, 50; NDF, 765: ADF, 454; ADL, 64; ash, 65; and GE 16.5 MJ/kg) and the second group received a roughage hay:concentrate diet (60:40; Ridley AgriProducts Pty Ltd, Brisbane, QLD, Australia. Concentrate ingredients (g/kg): barley (574), sorghum (200), molasses mixer (30), cotton hull pellet (100), urea (5); concentrate chemical composition: DM, 906 g/kg fresh matter; in g/kg of DM: OM, 906; CP, 116; NDF, 263; ADF, 120; ADL, 30; fat, 34; ash, 74; and GE 17.2 MJ/kg) being the hay the same for both diets. Animals were adapted to each diet over an initial 17 days period. After that initial period, experimental animals were maintained in individual pens in an animal house for the measurement of individual intakes (10 days) and were treated with cyclodextrin (CD; 3 g/100 kg LW). On days 9 and 10 animals were confined in open-circuit respiration chambers for measurement of CH_4_ and H_2_ production and collection of rumen samples (control period). Following the initial adaption/control period animals received a low dose of chloroform-cyclodextrin (CCD; 1 g/100 kg LW) for 10 days with the last 2 days being confined in open-circuit respiration chambers for direct measurement of CH_4_ and H_2_ production. Doses were then increased to a mid level (1.6 g/100 kg LW) for 10 days with rumen fluid collection and CH_4_/H_2_ measurements, and then to a high level (2.6 g/100 kg LW) for 10 days with a similar sampling regime for the final 2 days. The CCD doses were split up in two shots and administered through the rumen cannula at 0 and 3 h after feeding. After a 15 days period without CCD animals were returned to open circuit respiration chambers during two consecutive days with a similar rumen sampling regime (post-treatment period). Rumen fluid samples (60 mL per animal) were collected using a probe covered with two layers of cheesecloth at 3 h post feeding and just before dosing with CCD during each confinement period in respiration chambers. Rumen samples were stored at -20°C for short chain fatty acids (SCFA) and NH_3_-N analyses. Additionally, 20 mL were kept at -80°C for DNA extraction and metabolite analyses.

### Antimethanogen Formulation

The antimethanogen was an halogenated hydrocarbon (chloroform) entrapped in a β-CD matrix (May et al., 1995). The formulation was prepared by CSIRO Manufacturing (Clayton, VIC, Australia) in 3-kg batches and contained 6–7% w/w chloroform. Chloroform was encapsulated within a CD matrix, to increase its stability and slow its rate of release in the rumen. Similar halogenated analogs (such as bromochloromethane) entrapped in a CD matrix have been previously used in ruminants, to delay the release of the antimethanogenic compound in the rumen (Denman et al., 2007; Abecia et al., 2012; Mitsumori et al., 2012).

### Gas Measurements

Four open circuit respiration chambers were used to determine CH_4_ production from individual steers. Each chamber had an internal volume of 23.04 m^3^ and was equipped with a water trough and feed bin containing the daily ration. Each chamber was maintained at 2°C below ambient air temperature, approximately -10 Pa, and the relative humidity for the two, 24 h measurement periods varied from 50 to 75%. Air was drawn through a 250 mm diameter duct into each chamber at a rate of 3000 L/min. Exact flow rates, corrected to measured conditions for temperature and pressure for each chamber were used in calculations for CH_4_ and H_2_ production (Takahashi et al., 1999; Williams et al., 2007). Flow rate through each chamber was measured using thermal flow sensors (SS20.500 SCHMIDT^®^ Flow Sensor). The air sample for the analysis of gas composition was drawn from a point in the exhaust duct through polyurethane tubing at 4.5 L/min using a micro diaphragm pump located between a multiport gas switching unit (SW & WS Burrage, Ashford Kent UK) and membrane drier (Perma Pure LLC). Air samples from each chamber initially passed through particulate filters (AF30-02 SMC Pneumatics Aust. Pty Ltd) and a four port fridge drier prior to the multiport gas switching unit which was programmed to cycle through each chamber and two outside air ports. Air samples passed through a chemical drier and were metered through independent rotameters before compositional analysis for CO_2_ and CH_4_ (Servomex 4100 Servomex Group Ltd. Crowborough, UK) and H_2_ (Servomex Chroma, Servomex Group Ltd, Crowborough, UK; and Dräger X-am 5000, Draeger Safety Pacific Pty. Ltd., Notting Hill, VIC, Australia). Data for flow rate, temperature and chamber pressure, and CH_4_/H_2_ content of the exhaust air for the final 315 s of each sampling event was used to calculate CH_4_ and H_2_ flux.

### Chemical Analysis

The feed samples were dried in a forced-air oven at 105°C prior to grinding. Feed samples were ground through a 1-mm sieve before analysis. DM, ash, NDF, ADF, lignin, fat, gross energy (adiabatic calorimeter), and total nitrogen contents were analyzed by Symbio Alliance (Eight Mile Plains, QLD, Australia) following the accredited methods CF006.1, CF007, CF038.3, CF038.3, CF038.6, CF004.1, CF237, and CF003.2, respectively (Association of Official Analytical Chemists [AOAC], 2005 official methods: 925.60, 923.03, 920.39, 990.03, 2002.04, and 973.18). The nitrogen values were converted to CP by multiplying by 6.25.

Concentrations of SCFAs (acetate, propionate, *n*-butyrate, iso-butyrate, iso-valerate, and *n*-valerate) were measured by gas chromatography (GC) as described by Gagen et al. (2014). Iso-valerate (3-methyl butyrate) includes 2-methylbutyrate, which co-eluted.

The NH_3_-N concentration was determined by a colorimetric method following Chaney and Marbach (1962).

An UltiMate^®^ 3000 HPLC system (Dionex, Sunnyvale, CA, USA) with a dedicated Photodiode Array Detector and an Autosampler was used to determine the presence of formic acid in samples supernatants as described by Gagen et al. (2014).

### Calculation of [H] Redirection and Non-carboxyl SCFA Carbons

As actual flows of metabolites formation were not measured, concentrations were used as a proxy to estimate changes in the incorporation of [H] into SCFA (HUSr) and formate (HUFr). The stoichiometry was calculated as follows (Goel et al., 2009):

H⁢U⁢S⁢r=2⁢x⁢ C⁢3+2⁢x⁢ C⁢4+C⁢5

H⁢U⁢F⁢r=C⁢1

The CH_4_ gas production (GP; mol/day) and H_2_ gas production (GP; mol/day) was used to calculate the ratio between [H] redirected to H_2_ expelled/CH_4_ decrease ((H_2_ GP)/(CH_4_ GP decrease × 4)).

Total non-carboxyl SCFA carbon concentration were calculated using the following formula:

T⁢o⁢t⁢a⁢l⁢ n⁢o⁢n−c⁢a⁢r⁢b⁢o⁢x⁢y⁢1⁢ S⁢C⁢F⁢A⁢ c⁢a⁢r⁢b⁢o⁢n⁢ c⁢o⁢n⁢c⁢e⁢n⁢t⁢r⁢a⁢t⁢i⁢o⁢n=[A⁢C⁢L−1]⁢x⁢ m⁢M⁢ t⁢o⁢t⁢a⁢l⁢ S⁢C⁢F⁢A

Average chain length (ACL) = [mM Formate + (2x mM Acetate) + (3x mM Propionate) + (4x mM Butyrate) + (5x mMValerate)]/[Total mM Formate + Acetate + Propionate + Butyrate + Valerate]

The calculation removes from consideration the carboxyl group of the SCFA and provides a standard for comparison for SCFA energy available to the animal (Ungerfeld, 2013).

The non-carbohydrate contributions to SCFA were assumed to be unimportant and were not considered in the calculations described above.

### Rumen Metabolomics Analyses

Samples from rumen fluid were prepared and metabolites quantified by Metabolomics Australia, University of Melbourne.

Amines quantification: Sample volumes of 10 μL of rumen fluid supernatant were placed in 2 mL Eppendorf tubes under cold conditions (4°C). Methanol (100% MeOH, 250 μL) containing four internal standards [^13^C-sorbitol (0.5 mg/mL), ^13^C_5_-^15^N-Valine (0.5 mg/mL), 2-aminoanthracene (0.25 mg/mL), and pentafluorobenzoic acid (0.25 mg/mL)] was added to the sample tubes and the samples were vortexed for 5 min until uniform. The samples were then incubated in a Thermomixer (Eppendorf brand, distributed by Quantum Scientific, Australia) at 70°C with a mixing speed of 850 rpm for 15 min, followed by a 15 min of centrifugation at 4°C at 13,800 × *g* in an Eppendorf benchtop centrifuge. The MeOH supernatant was transferred into a new 2 mL Eppendorf tube and set aside. Water (250 μL, Milli-Q grade) was added to the remaining sample pellet in the initial tube and vortexed for 5 min before being centrifuged at 13,800 x *g* at 4°C for 15 min. The H_2_O supernatant was transferred to the Eppendorf tube containing the MeOH supernatant and vortexed to mix. A 10 μL aliquot was transferred to a fresh Eppendorf tube in preparation for derivatisation with 6-aminoquinolyl-*N*-hydroxysuccinimidyl carbamate (Aqc) followed by LC-MS analysis as per Boughton et al. (2011). In summary, 70 μL borate buffer was added to the sample aliquot, vortexed to mix and then centrifuged at 13,800 × *g* at 4°C for 1 min. Then, 20 μL Aqc reagent was added to the sample aliquot, vortexed immediately to mix and then centrifuged at 13,800 × *g* at 4°C for 1 min. The Aqc-treated samples were incubated in a Thermomixer at 55°C with a mixing speed of 1150 rpm for 10 min and then centrifuged at 13,800 × *g* at 4°C for 5 min. The derivatized samples were then transferred to HPLC vials for LC-MS analysis as described by Boughton et al. (2011).

Sugars, organics, and fatty acids quantification: Sample volumes of 240 μL of rumen fluid supernatant were placed in 2 mL Eppendorf tubes under cold conditions (4°C) and 320 μL of 100% methanol, containing 2% ^13^C_6_-Sorbitol as a quantitative internal standard, was added. The samples were then vortexed for 1 min and incubated with shaking (950 rpm) at 30°C for 15 min prior to centrifugation at 13,800 × *g* for 15 min at room temperature in a bench-top Eppendorf centrifuge. The supernatant was transferred to a clean tube while the remaining pellet was re-extracted with 640 μL of CHCl_3_ and vortexing for 1 min. After re-centrifugation as before, 60 μL of the polar upper phase was removed and dried under vacuum without heating prior to preparation for sugars and organic acids quantitation as described by Dias et al. (2015). The lower CHCl_3_-phase was dried under vacuum without heating then reconstituted in 320 μL of CHCl_3_ before a 160 μL aliquot transferred to a glass insert and re-dried under vacuum prior to preparation for fatty acid quantitation described by Dias et al. (2015).

### DNA Extractions

DNA extractions were carried out on rumen samples using the cetyltrimethylammonium bromide (CTAB) method of Brookman and Nicholson (2005) with minor modifications as follows: samples were centrifuged (13,000 × *g* for 5 min), and the supernatant was removed before DNA extraction. Cells were homogenized with 200 mg of silica–zirconium beads (1:1 mixture of 0.1- and 1.0-mm beads; Biospec, Bartlesville, OK, USA) and 800 μl of CTAB buffer in a Mini-Beadbeater-8 (Biospec) on maximum speed for 2 min, twice. Samples were incubated at 70°C for 20 min and centrifuged at 10,000 × *g* for 10 min, and the supernatant was mixed with 500 μl of 25:24:1 phenol–chloroform–isoamyl alcohol (Fluka BioChemika, Buchs, Switzerland). The yield and purity of the extracted DNA were assessed with a NanoDrop 8000 spectrophotometer (Thermo Fisher Scientific, Wilmington, DE, USA).

### Real-Time PCR Analysis

The DNA samples were used as templates for quantifying the abundance of anaerobic rumen fungi, protozoa populations, and mcrA gene for methanogens. The primers and assay conditions used were previously published (Sylvester et al., 2004; Denman and McSweeney, 2006; Denman et al., 2007). Real-time PCR (qPCR) analyses were run in triplicate from one DNA extraction on an Applied Biosystems^TM^ ViiA^TM^ 7 Real-Time PCR System (Thermo Fisher Scientific Inc.). Assays were set up using the SensiFAST SYBR^®^ Lo-ROX (Bioline). Optimisation of assay conditions was performed for primer, template DNA, and MgCl_2_ concentrations. An optimal primer concentration of 400 nM, a final MgCl_2_ concentration of 3 mM and DNA template concentration of 50 ng were used for each assay under the following cycle conditions: one cycle of 50°C for 10 s and 95°C for 2 min 30 s for initial denaturation, forty cycles at 95°C for 15 s and 60°C for 1 min for primer annealing and product elongation. Fluorescence detection was performed at the end of each annealing and extension step. Amplicon specificity was performed via dissociation curve analysis of PCR end products by raising the temperature at a rate of 0.05°C/s from 60 to 95°C. Changes in targeted populations were calculated using a relative quantification calculation and the 2^-ΔΔC_*t*_^ method, with the control period used as the calibrator and total bacterial (Denman and McSweeney, 2006) C_t_ (cycle threshold) values used as the reference value (Livak and Schmittgen, 2001). Estimation of abundance of target populations to indicate their contribution to the sample was also calculated using standard curves for each target gene generated from cloned PCR products (Mitsumori et al., 2012).

### 16S rDNA Analysis

Using high throughput sequencing platforms and barcoded primer sets, phylogenetic based methods targeting the 16S rDNA gene were used to deeply characterize the microbial populations present in the rumen for the control and treatment periods. The V4 region of the 16S rRNA gene was targeted using specific primers (Kozich et al., 2013). Each individual DNA sample was amplified using the specific primers and a unique barcode combination. Afterward, amplification products were visualized by performing gel electrophoresis. Product quantities were calculated and an equal molar amount of each product was pooled. The pooled products were run in a 1% agarose gel and bands were visualized and excised under blue light transillumination. The amplicons were gel purified with QIAquick Gel extraction Kit (Qiagen, Hilden, Germany) prior to submission for Illumina Miseq.

Short read sequence data generated was analyzed using QIIME: Quantitative Insights Into Microbial Ecology software package (Caporaso et al., 2010). Sequences were clustered as operational taxonomic units (OTUs) of 97% similarity using uclust (Edgar, 2010). Taxonomic assignment of sequences was performed against the Greengenes database (McDonald et al., 2012). Alpha and beta diversity and significant fold change of OTU’s were performed in the R packages ade4, Phyloseq, and DESeq2 (Chessel et al., 2004; McMurdie and Holmes, 2013; Love et al., 2014). The sequences obtained in this paper have been deposited in the European Nucleotide Archive (ENA) under the accession number PRJEB13653.

### Statistical Analyses

The effect of dose, diet, and their interaction were analyzed for the CH_4_/H_2_ production, DMI and fermentation variables as a univariate repeated-measures analysis of variance using the GLM procedure of SPSS (IBM, version 21.0), with animal as the experimental unit. Linear and quadratic components of the response to incremental dose of CCD were evaluated using polynomial contrasts. Effects were considered significant at *P* ≤ 0.05 and considered as tendencies toward significance at *P* ≤ 0.10. When significant differences were detected, differences among means were tested by pairwise comparisons (LSD test). The model used was:

$$Y\,i\,j\,t\,k=\mu +d_{j}+f_{k}+{\left(d\times f\right)}_{j\,k}+\varepsilon_{i\,j\,t\,k}$$

Where:

y_ijtk_ is the dependent variable measured at time t on the i-th steer treated with the j-th dose and the k-th feed,

μ the overall mean,

d_j_ is the fixed dose effect,

f_k_ is the fixed feed effect,

(d × f)_jk_ the interaction between dose and feed,

𝜖_ijkt_ the residual error associated with the i-th steer within the j-th dose and k-th feed at time t.

## Results

### Ruminal Fermentation and Gas Production

There was an interaction (*P* = 0.040) between dose and diet on DM intake (DMI), with a decrease in DMI with CCD dose increase in the hay:concentrate diet, and an increase in DMI with CCD dose increase in the hay diet (**Table 1**). Methane production (g/kg DMI) was decreased significantly (*P* < 0.01) for mid and high doses compared with the control on both diets. Conversely H_2_ expelled by treated animals increased significantly (*P* < 0.05) as CH_4_ production was decreased, with the greatest amounts of H_2_ release (g/kg DMI) occurring in animals supplemented with the roughage hay:concentrate diet. Both effects were linear, showing a dose-dependent response. In addition the amount of H_2_ expelled relative to the decrease in CH_4_ (mol H_2_/mol CH_4_ decrease) was greater in animals fed the concentrate diet showing a significant (*P* < 0.05) diet effect, although no diet–dose interaction was observed. Fourteen days after the CCD treatment was terminated, the CH_4_ and H_2_ production were no significantly different to the control period for both diets (data not shown).

**Table 1:** Control and CCD doses (low, mid, and high) effects on DMI, CH_4,_ and H_2_ production, and rumen fermentation variables from samples collected 3 h after feeding of animals fed with hay:concentrate or hay diet.

	Hay:concentrate diet				Hay diet						*P*-value			
Item	Control	Low	Mid	High	Control		Low	Mid	High	SEM	Dose	Diet	Dose × Diet	Contrast
DMI kg	5.9	5.8	5.6	5.0	4.2		4.6	4.4	4.7	0.28	0.494	0.005	0.040	
CH_4_ (g/kg DMI)	24^a^	19^b^	15^bc^	10^bc^	27^a^		21^b^	17^bc^	12^bc^	1.40	0.001	0.160	0.998	*L*
H_2_ (g/kg DMI)	0.00^d^	0.97^c^	1.43^b^	3.16^a^	0.00^c^		0.33^b^	0.83^a^	1.73^a^	0.18	0.001	0.029	0.144	*L*
mol H_2_/molCH_4_ decrease^1^	–	1.7	1.4	1.6	–		0.66	0.63	0.89	0.41	0.813	0.020	0.898	
pH	6.4^b^	6.6^a^	6.6^a^	6.7^a^	6.6^c^		6.7^ab^	6.8^a^	6.7^b^	0.04	0.001	0.026	0.010	*L, Q*
Formate (mM)	0.0^d^	4.2^c^	8.5^b^	12^a^	0.0^b^		0.0^b^	0.0^b^	4.2^a^	1.06	0.001	0.001	0.001	*L*
NH_3_–N (mg/100 mL)	4.4	2.8	3.1	4.2	2.3		4.7	4.8	5.2	0.54	0.337	0.137	0.063	
Total SCFA (mM)	88^a^	81^ab^	79^ab^	78^b^	79^b^		91^a^	82^ab^	66^c^	2.99	0.001	0.616	0.005	*L, Q*
Fatty acid (%)														
Acetate	62^a^	56^b^	55^b^	53^b^	67^a^		62^b^	61^b^	58^c^	1.31	0.001	0.002	0.575	*L, Q*
Propionate	18^b^	21^a^	21^a^	23^a^		15^b^	18^a^	18^a^	19^a^	0.76	0.001	0.001	0.973	*L*
Butyrate	12^b^	13^a^	14^a^	13^a^		9^b^	11^a^	11^a^	12^a^	0.61	0.019	0.011	0.745	*L, Q*
i-Butyrate	1.53^b^	1.74^a^	1.81^a^	1.89^a^		1.69^c^	1.66^c^	1.85^b^	2.13^a^	0.04	0.001	0.222	0.004	*L*
Valerate	3.45^b^	3.98^a^	4.03^a^	4.07^a^		3.56^c^	3.76^bc^	4.01^b^	4.69^a^	0.10	0.001	0.488	0.006	*L*
i-Valerate^2^	3.05^c^	3.80^b^	4.39^a^	4.69^a^		3.15^c^	3.31^c^	3.92^b^	4.79^a^	0.15	0.001	0.522	0.062	*L*
A:P	3.44^a^	2.65^b^	2.60^b^	2.34^b^		4.41^a^	3.50^b^	3.41^b^	3.02^c^	0.20	0.001	0.002	0.793	*L, Q*
Non-carboxyl SCFA carbon	135^a^	125^ab^	117^b^	112^b^		116^b^	140^a^	131^a^	99^c^	4.39	0.002	0.854	0.010	*L, Q*

*^1^Taken into account the DMI per animal. Moles of H_2_ and CH_4_ were calculated from the expelled gases.*

*^2^I-valerate (3-methyl butyrate) includes 2-methylbutyrate, which co-eluted.*

*SEM, standard error of the mean.*

*^a-d^Within a row treatment means for each diet without a common superscript differ, P < 0.05.*

*Contrast: Significant (P < 0.05) linear (L) or quadratic (Q) effects of the response to incremental dose of CCD estimated by polynomial contrast.*

Rumen SCFA analysis showed a shift in the fermentation pathways (**Table 1**) toward a higher propionate profile when CCD was linearly increased on both diets. This was reflected by a significant decrease in acetate molar percentage (*P* < 0.001) and an increase in propionate molar percentage (*P* < 0.05) for both diets. As a result, a significant linear and quadratic decrease of the acetate:propionate ratio was observed at all doses for both diets. A diet–dose interaction (*P* = 0.005) was observed for the total SCFA concentration, with a significant decrease with the highest dose of CCD for the hay:concentrate diet. The hay diet, however, showed a significant (*P* < 0.001) increase in total SCFA concentration when animals were treated with the low and mid dose of CCD and a decrease with the high dose. Butyrate molar percentage linearly increased with CCD with both diets (*P* < 0.011). The branched-chain SCFA linearly increased with both diets, showing a diet–dose interaction effect (*P* < 0.05), with the greatest increase observed for the hay diet. Rumen pH increased linearly with CCD (*P* < 0.001) treatment for both diets showing a diet–dose interaction (*P* = 0.01). Rumen ammonia concentration was unchanged compared with the control period for both diets. A significant linear increase in formate concentration was observed for all CCD doses for the hay:concentrate diet and only with the hay diet at the highest CCD dose, showing a diet and a diet–dose interaction (*P* < 0.001). Regarding the diet effect, it was observed for formate, pH, acetate, propionate, butyrate, and acetate:propionate ratio. Interestingly, the total non-carboxyl SCFA carbons linearly decreased (*P* = 0.002) for the hay:concentrate diet and increased for the hay diet at low and mid doses, showing a diet-dose interaction (*P* = 0.01).

Rumen metabolite profiles for control and treated animals at mid dose of CCD are shown in Supplementary Table S1. Some amino acids, organic acids and sugars were significantly increased on both diets in animals treated with CCD, although the profile observed was different for each diet. A greater number of metabolites increased significantly (*P* < 0.05) for those animals fed the hay diet, (amino acids: serine, homoserine, asparagine, threonine, proline, valine, isoleucine, leucine, tyramine, and phenethylamine; the organic acids: malate, fumarate, malonate, and nicotinic acid; and the sugars: arabitol, fructose, and inositol). In animals fed the hay:concentrate diet the greatest increase (*P* < 0.05) was observed for the amino acids: homoserine, asparagine, proline, valine, isoleucine, glutamate (*P* = 0.053), and leucine (*P* = 0.080); and the sugars: ribose, arabitol, and inositol. A marked fold increase was observed in nucleic acid precursors/derivatives (inosine and hypoxanthine) in CCD treated animals compared with the control group, fed with hay (*P* ≤ 0.05) or hay:concentrate (*P* ≤ 0.10) diet, while no significant effect was observed on lactate concentrations (Supplementary Table S2).

**FIGURE 1:**
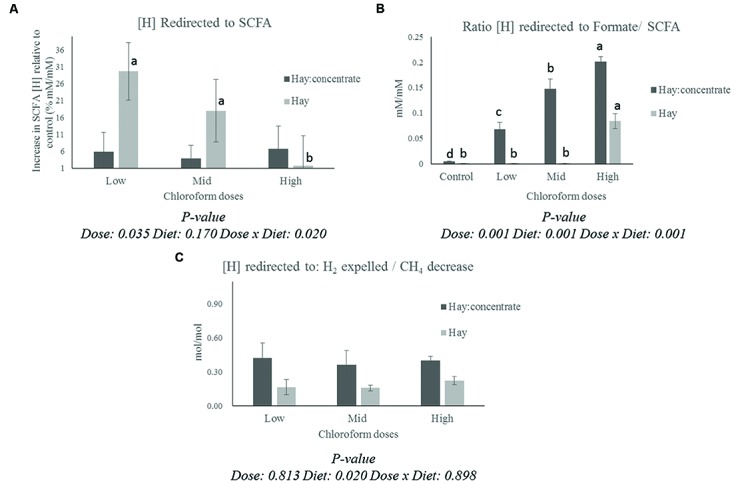
**Effect of CCD doses on (A) redirection of [H] to SCFA (Increase of [H] incorporated into SCFA relative to control for each diet, % mM/mM), (B) the ratio between [H] incorporated into formate and [H] incorporated into SCFA for each diet, and (C) the ratio between [H] redirected to H_2_ expelled/CH_4_ decrease with both diets.** ^a-d^Letters denote significant differences between doses for each diet, bars that do not share the same letter are significantly different from each other in each diet (*P* < 0.05).

The calculation of the [H] redirection showed a different pattern for the [H] into SCFA (**Figure 1A**) and formate/SCFA ratio (**Figure 1B**) for each diet. All the CCD doses showed a significant (*P* ≤ 0.001) increase in [H] redirected into formate for the hay:concentrate diet, while for the hay diet, a measurable proportion of [H] was recovered in formate only at the highest CCD dose. On the other hand, a significant increase (*P* ≤ 0.05) of [H] redirected into SCFA was observed with the low and mid dose for the hay diet, while no significant effect was detected for the grain:concentrate diet. Interestingly, a diet effect (*P* ≤ 0.05) was observed for the H_2_/CH_4_ decrease ratio (**Figure 1C**), being more [H] recovered into H_2_ per mole of CH_4_ decrease for the hay:concentrate compared with the hay diet.

### Microbial Community

The dose effect of CCD on the abundance of methanogens, protozoa, and fungi are shown in Supplementary Figure S1 for hay:concentrate and hay diet, respectively. The methanogen abundance decreased (*P* < 0.05) with increasing doses of CCD for both diets. The CCD doses tended to increse the protozoa abundance in animals fed with the hay:concentrate diet, whereas the anaerobic fungi were not significantly affected by the CCD doses for either diets.

The diversity analysis of the rumen microbiota showed that total microbial species richness was impacted with the administration of all CCD doses in animals fed the hay:concentrate diet, whilst only the highest dose of CCD resulted in a significantly altered for the hay diet. While the CCD caused an increase in observed and estimated speciess richness for hay:concentrate diet animals, a contraction in Shannon diversity was observed for mid and high doses on a roughage diet. The microbial species richness from the post-treatment period samples showed similar values to the highest dose of CCD on both diets (Supplementary Figure S2).

The composition of the microbiomes as determined by beta diversity analysis showed a clear separation between the control and CCD doses for both diets (Supplementary Figure S3). The post-treatment period was similar to the low CCD dose or intermediate between that dose and the control treatment. The greatest variance observed between control and CCD groups was 18 and 15% in hay:concentrate and roughage diets, respectively. Variation between animals explained the next level of variance irrespective of treatment due to one animal possessing a different microbial population compared to the other three animals on both diets. The variance between individual animals decreased at the high CCD dose and post-treatment period (Supplementary Figure S3).

**FIGURE 2:**
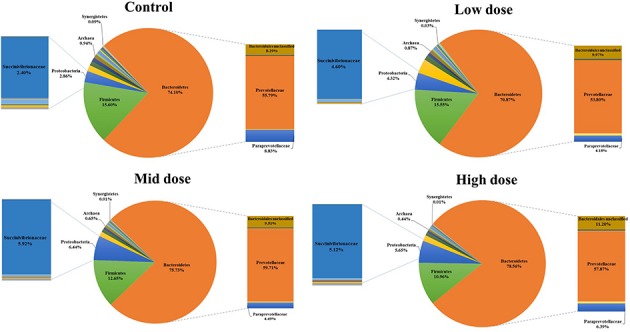
**Taxonomic composition of rumen microbiome at the phylum level (pie chart) and family level (bar chart) for control and CCD (low, mid, and high doses) in animals fed with hay:concentrate diet 3 h after feeding**.

**FIGURE 3:**
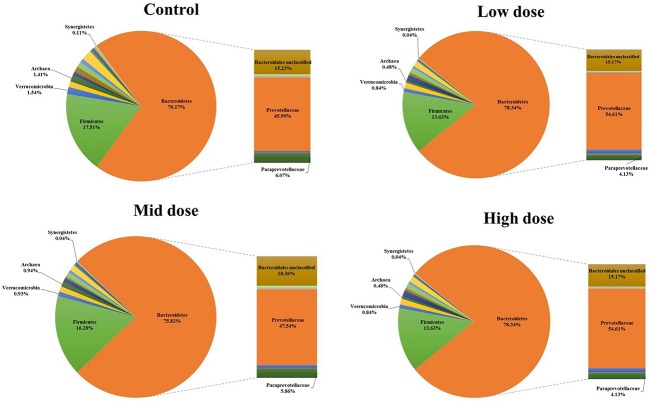
**Taxonomic composition of rumen microbiome at the phylum level (pie chart) and family level (bar chart) for control and CCD (low, mid, and high doses) in animals fed with hay diet 3 h after feeding**.

**FIGURE 4:**
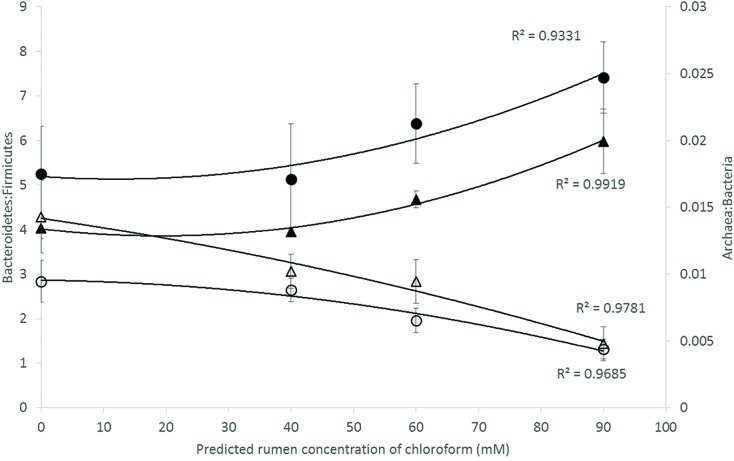
**Microbial ratios [Bacteroidetes:Firmicutes (B:F) and Archaea:Bacteria (A:B)] for increasing concentration of chloroform on animals fed with hay [B:F (▲) or A:B (△)] or hay:concentrate [B:F (●) or A:B (◯)] diet 3 h after feeding.** Predicted rumen concentration of chloroform: 40, 60, and 90 μM for low, mid, and high dose, respectively.

**FIGURE 5:**
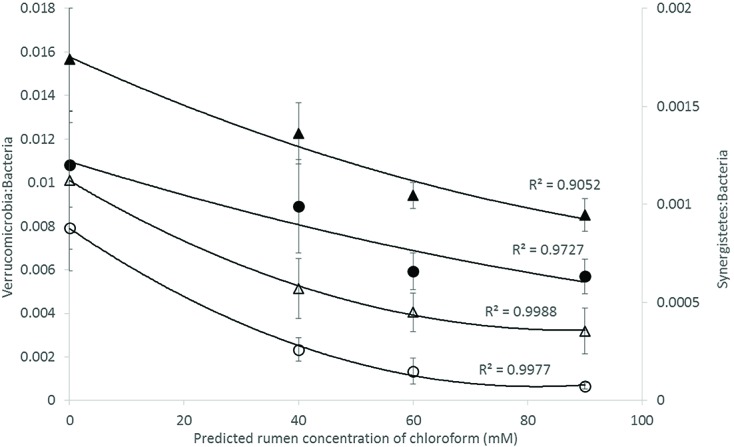
**Microbial ratios [Synergistetes:Bacteria (S:B) and Verrucomicrobia:Bacteria (V:B)] for increasing concentration of chloroform on animals fed with hay [V:B (▲) or S:B (△)] or hay:concentrate [V:B (●) or S:B (◯)] diet 3 h after feeding.** Predicted rumen concentration of chloroform: 40, 60, and 90 μM for low, mid, and high dose, respectively.

Analysis of the rumen microbiome showed a shift in the relative abundance at the phylum level when the CCD dose was increased in both diets. An increase in the sequences assigned to the Bacteroidetes phylum and a decrease in Firmicutes, Synergistetes, Verrucomicrobia, and Archaea were observed in both diets (**Figures 2** and **3**). An increase in sequences assigned to the Proteobacteria phylum, mainly classified as Succinivibrionaceae family, were observed in the hay:concentrate diet animals upon treatment with CCD in contrast with hay diet. Consequently the Bacteroidetes:Firmicutes ratio increased with both diets when methanogenesis was inhibited (**Figure 4**). The ratios of sequences assigned to Archaea, Synergistetes, and Verrucomicrobia in relation to bacteria, decreased when CCD dose increased and methane was inhibited showing a dose-dependent effect (**Figures 4** and **5**).

Specific OTUs that were significantly increased with CCD treatment were classified in the *Prevotella* genus, for both diets. Specifically for the animals fed with the hay:concentrate diet, a few OTUs that increased in abundance with CCD were assigned to Moraxellaceae and Succinivibrionaceae family in the Proteobacteria phylum, and the fiber degrading microorganisms *Fibrobacter succinogenes* and *Ruminococcus* spp. Regarding the roughage hay fed animals, minor OTUs promoted by the CCD doses were within the Paraprevotellaceae family and *Butyrivibrio* genus for all the doses and at the highest dose, respectively (Supplementary Figures S4–S9).

The increasing level of CCD was negatively associated in both diets with the abundance of OTUs assigned to *Prevotella* genus compared with the control, which could suggest a shift within the Prevotella groups through both diets (Supplementary Figures S4–S9). In relation to the fibrolytic microorganisms minor OTUs assigned to *Fibrobacter* genus were decreased in both diets with the lowest dose but did not change or were promoted with the increasing doses of CCD, which could suggest a shift in those populations. OTUs assigned to the fibrolytic species *R. albus* did not change with the doses, although a single OTU classified as *R. flavefaciens* was suppressed at the highest dose of CCD with the hay diet.

The Archaea domain was negatively affected by CCD levels in both diets in accordance with the decrease observed in the Archaea:Bacteria (A:B) ratio. Specific OTUs assigned to the Methanobacteriaceae family and Methanoplasmatales order were decreased by the CCD levels (Supplementary Figures S4–S9).

## Discussion

This study established a model in cattle whereby methanogens were directly inhibited in a dose dependent manner and the subsequent responses in rumen microbial metabolism were evaluated. The three levels of CCD, low, medium, and high, decreased CH_4_ production by approximately 14, 37, and 55%, respectively, on average of both diets compared to the control period with no apparent effect on feed intakes. Conversely H_2_ expelled by treated animals showed a dose-dependent increase as CH_4_ decreased. A similar inverse relationship between CH_4_ decrease and H_2_ loss has been reported in dairy cows treated with nitrate or 3-nitrooxypropanol, and goats fed the CH_4_ inhibitor BCM (Mitsumori et al., 2012; Hristov et al., 2015; Veneman et al., 2015). However, importantly this study showed that greater amounts of H_2_ (1.7- to 2.9-fold; g/kg DMI) were expelled in animals supplemented with the hay:concentrate diet compared to the hay only diet. Furthermore the amount of H_2_ expelled in the CH_4_ inhibited animals was lower than the predicted amount of H_2_ involved in hydrogenotrophic CH_4_ formation (four moles of H_2_/mole CH_4_). This suggests that significant amounts of [H] were redirected into reduced end products other than CH_4_ and H_2_, and perhaps microbial protein in agreement with the meta-analysis of Ungerfeld (2015). Interestingly the rumen microbiota in the hay-fed animals appeared to utilize more H_2_ that was available from the decrease in CH_4_ formation than their hay:concentrate-fed counterparts, possibly due to the slower fermentation rate of the hay diet compare with the highly fermentable hay:concentrate diet which might produce a more consistent release of hydrogen. Two *in vitro* studies (Lin et al., 2013; O’Brien et al., 2013) observed a greater accumulation of H_2_ when inhibiting methanogenesis in mixed roughage concentrate fermentations compared with the roughage substrates. Furthermore, a recently published article by Vyas et al. (2016) also shows increased expulsion of H_2_ for methanogenesis-inhibited animals fed a high concentrate diet compared with a mixed forage:concentrate diet.

In relation to the redirection of [H]_,_ on both diets there was a shift in fermentation from acetate to fatty acids that were longer in length, particularly propionate that is a major gluconeogenic precursor in ruminants (Newbold et al., 2005). This pattern of fermentation along with an increase in branched chain fatty acids, has been reported previously in studies using the halogenated methane analog, BCM, as the methane inhibitor (Denman et al., 2007; Abecia et al., 2012; Mitsumori et al., 2012). In fact, the total non-carboxyl SCFA carbons increased at the low and mid dose with the hay diet, which might indicate that [H] is more effectively redirected into SCFA for that diet. The rumen microbiome analysis showed specific OTUs assigned to the *Prevotella* genus were promoted when methanogenesis was inhibited. Some of these *Prevotella* OTUs promoted by chloroform with the hay:concentrate diet (results not shown), were closely associated with the *Prevotella* group 7 which was increased in goats inhibited with BCM (Mitsumori et al., 2012). *Prevotella* species appear to increase propionate production via the randomizing pathway when methanogenesis is inhibited (Denman et al., 2015). The *Prevotella* OTUs, promoted in the CCD treated animals, may occupy the niche vacated by those *Prevotella* OTUs that declined as a result of increasing CCD concentration. OTUs assigned to the *Butyrivibrio* genus were positively affected by CCD in roughage fed animals, which might be a contributor to the increased butyrate in these animals.

Another potential [H] sink in absence of methanogenesis is acetate produced from reductive acetogenesis (Fonty et al., 2007). In the present study, the acetic acid concentration decreased with CCD treatments, which possibly indicates that reductive acetogenic bacteria have not contributed significantly to the redirection of [H]. However, it has been observed that chloroform might inhibit acetogens (Knight et al., 2011). The notion that acetogens would be promoted through an increase in availability of [H] in the methanogenesis-inhibited rumen remains unresolved and may be confounded by using chemical inhibitors that target methanogenesis but may inhibit reductive acetogenesis.

A particularly interesting observation was the gradual increase in formate concentration as methane formation declined and hydrogen accumulated. This has been observed previously when CH_4_ analogs such as chloroform inhibited methanogenesis (Thiele and Zeikus, 1988). A meta-analysis of studies involving methane inhibition also identified that increased formate was a characteristic response to methane inhibition (Ungerfeld, 2015). Leng (2014) suggested that formate accumulates when methanogenesis is inhibited, and this helps to maintain a steady partial pressure of H_2_ in the rumen fluid. Formate is produced by ruminal bacteria and fungi and it is mainly consumed by specific methanogens as a precursor for CH_4_ formation (Hungate et al., 1970; Bauchop and Mountfort, 1981; Asanuma et al., 1998). Leng (2014) cited studies showing that some methanogens can produce formate when inhibited with halogenated hydrocarbons (Thiele and Zeikus, 1988; Bleicher and Winter, 1994). It is possible therefore that the increase of formate concentration might be due to a balance between an increase in production and a decrease in utilization, when methanogenesis is inhibited. Furthermore, formate has a greater coefficient of diffusion compared to H_2_ (Boone et al., 1989). Thus, we hypothesize that more reducing equivalents might be released through pyruvate formate liases compared to pyruvate oxidoreductases when H_2_ accumulates. Formate might help in the control of H_2_ partial pressures in the rumen, playing a role as hydrogen sink and being an indicator of H_2_ partial pressure. This is in accord with the present study where methanogenesis-inhibited animals fed with the hay:concentrate diet had higher concentrations of formate and H_2_ release than those only fed with the roughage hay.

A shift in SCFA pattern and increase in formate were not the only significant changes, other metabolites such as amino acids and nucleic acids increased in rumen fluid as H_2_ concentration rose. There were increases in amino acids and nucleic acids, which could be indicative of an increase in proteolysis and microbial growth. In our study, metabolites (such as hypoxanthine, inosine, or nicotinic acid) which are degradation products of microbial cells and diet, increased with CCD treatment. Nucleic acids can breakdown into inosine, xanthine, hypoxanthine, and uracil, and nicotinic acid can increase microbial protein synthesis (McAllan and Smith, 1973; Riddell et al., 1980). The amino acid profile showed an increase in valine, leucine, and isoleucine that could be due to greater digestion of protein in the rumen. Another important amino acid which increased when methanogenesis was inhibited, was aspartate. This amino acid is the transamination product of oxaloacetic acid which is produced in the succinate–propionate (randomizing) pathway that is considered as a major route for propionate synthesis in the rumen (Baldwin et al., 1963; Joyner and Baldwin, 1966). An abundance of genes assigned to this pathway were found to be increased and predominately associated with *Prevotella* species in goats administered with the anti-methanogenic BCM (Denman et al., 2015). Other intermediates of the randomizing pathway, such as malate and fumarate, increased when methanogenesis was inhibited particularly with the roughage diet, supporting the redirection found toward propionate. The increase in amino acids in the rumen may be due to proteolytic activity associated with the relative increase in Bacteroidetes and *Prevotella*-related bacteria. Ungerfeld (2015) suggested that inhibition of methanogens, could stimulate amino acids and fatty acids synthesis and therefore the increase in microbial biomass would also be a [H] sink under these conditions. Also, some studies (Russell and Jeraci, 1984; Russell and Martin, 1984; Hino and Russell, 1985) have shown that methanogenesis suppression resulted in inhibition of deamination of amino acids. This might be due to the antimethanogenic compound used (ionophores and hydrogenase inhibitors) and the extent of methane decreased (almost total suppression). On the other hand, later *in vitro* and *in vivo* studies using halogenated compounds (BCM) or a more specific antimethanogenic compound (3-NOP) showed consistently an increase on branched-chain fatty acids when methane was decreased (between 30 and 50%), which might suggest that deamination of amino acids was not negatively affected (Denman et al., 2007; Mitsumori et al., 2012; Martinez-Fernandez et al., 2014, 2015; Romero-Perez et al., 2014; Haisan et al., 2016). These observations are further supported by the metabolomic data in the present study, which showed an increase in more reduced metabolites and likely greater fermentation of amino acids as shown by increasing concentration of isoacids with increasing CCD dose, particularly in the hay fed animals.

The Bacteroidetes:Firmicutes (B:F) ratio (~75:17) observed in the untreated animals for both diets was substantially higher than has been reported in many other ruminant studies where temperate diets are common. Our study involving tropical adapted cattle fed a basal diet of tropical hay is in agreement with the high ratio reported by McCann et al. (2014) in Brahman cattle fed Coastal Bermuda-grass. In this study, a further increase in this ratio was observed in both diets when methane was decreased and is consistent with the redirection of hydrogen in the rumen. The Bacteroidetes are considered net H_2_ utilizers whereas the Firmicutes phylum contains a higher number of known H_2_ producers (Stewart et al., 1997). The same B:F shift was observed when methane was decreased *in vivo* and *in vitro* when methanogenesis was inhibited with BCM (Denman et al., 2015; Martinez-Fernandez et al., 2015). Cattle fed hay:concentrate, had a significant population of Proteobacteria (~3%) compared with the hay only diet and within this phylum, the predominant family Succinivibrionaceae which are involved in propionate production, increased with the three levels of CCD. A higher abundance of this family in low-emission beef cattle compared with high emitters has also been observed (Wallace et al., 2015).

Bacteria affiliated with the Synergistetes and Verrucomicrobia phyla were negatively correlated with the increasing levels of CCD and increases in H_2,_ for both diets. A similar negative relationship for these groups of bacteria and increased of H_2_ expelled has been previously reported in ruminants treated with BCM (Denman et al., 2015). In high and low methane emitting animals, Synergistetes were significantly more abundant in high emitters and there was a tendency for Verrucomicrobia to also be higher in these animals as well (Wallace et al., 2015). Collectively these data may indicate that Synergistetes and Verrucomicrobia bacteria are sensitive indicators of H_2_ partial pressures in the rumen fluid. Recently, Leong et al. (2016) demonstrated that interspecies H_2_ transfer between Synergistetes and methanogens enhanced the growth of the bacterium which may explain why these bacteria declined in abundance when methanogens were inhibited in the current study.

In relation to the rumen archaeal community, the A:B ratio decreased with increasing levels of CCD, which represented a fivefold to sevenfold decrease in the methanogen population and a 30–50% decrease in methane production. Furthermore the rumen microbiome analysis revealed that OTUs assigned to Methanobacteriaceae family and Methanoplasmatales order decreased with the increasing doses of CCD. Our results are in agreement with previous studies which observed a similar decrease using halogenated compounds to inhibit enteric methanogenesis in cattle (Denman et al., 2007). Mitsumori et al. (2012) observed that only a half-log reduction in the methanogen population was correlated with 50% decrease in methane when BCM was used. Previous studies (Wallace et al., 2015; Roehe et al., 2016) have also reported a higher proportion of A:B ratio from high methane emitting animals compared with low emitters. The decreasing A:B ratio observed when methanogenesis was inhibited may provide a simple index for relative methane production in ruminants.

It is assumed that H_2_ accumulation in the rumen impairs fiber digestion and therefore would reduce productivity (Wolin et al., 1997; Janssen, 2010). However, a previous study showed that inhibition of methanogenesis by BCM in small ruminants dramatically increased H_2_ without affecting DMI and feed digestibility (Mitsumori et al., 2012). In the present study, dry matter intakes were not affected and OTUs assigned to the fibrolytic species *R. albus* and *R. flavefaciens* did not change through the CCD low and mid doses compared with control period. Importantly, OTUs classified as *F. succinogenes* were positively associated with CCD in the hay:concentrate diet and were only negatively associated with the lowest dose in both diets. It is known that *F. succinogenes* might not be affected by H_2_ accumulation (Wolin et al., 1997). In fact, *F. succinogenes* populations increased in the presence of H_2_ that accumulated when BCM was used to inhibit methanogenesis but surprisingly *R. albus* abundance was unaffected even though it produces large amounts of H_2_ in the presence of methanogens (Mitsumori et al., 2012). Our results suggest that the bacterial fibrolytic community was not affected by the increasing level of H_2_, although the relative abundance of some particular OTUs changed. Further analyses to study how those changes affect the fiber degradability in the rumen should be carried out in future experiments.

Protozoa and fungi also play a key role in fiber degradation and rumen metabolism, and can be affected by the H_2_ accumulation in the rumen. No significant changes were observed in the abundance of those populations that suggests, in accordance with the microbial, metabolite and fermentation profiles, a non-detrimental effect on fiber digestion, and microbial and dietary proteolysis. However, further analyses using in-depth sequencing technology could be developed in future experiments to understand these important rumen community members.

## Conclusion

The present study in cattle showed that a decrease in methane formation by 30–35% resulted in a redirection of [H] into more reduced microbial end-products and eructation of excess H_2_ without an apparent adverse effect on DM intake, fibrolytic activity and general rumen function. The amount of expelled H_2_ per mol of decreased methane was lower for the hay diet suggesting a more efficient redirection of [H] perhaps due to the slower fermentation rate and evolution of H_2_ compared with the hay:concentrate supplemented animals. The metabolomics analysis showed increases in amino acids and nucleic acids concentration that may indicate an enhanced of the proteolysis and microbial protein synthesis in the rumen, particularly in the methanogenesis-inhibited cattle fed hay. These changes in metabolism were accompanied by a shift in the microbiota toward more Bacteroidetes and a decrease in Archaea and Synergistetes for both diets. Synergistetes among other bacteria groups, may be sensitive indicators of H_2_ partial pressures in the rumen. Although there was a redirection of [H], dietary supplements or microbial treatments might be needed to drive the excess H_2_ into energy-yielding substrates and consequently improve the energy supply to the animal.

## Author Contributions

CM, GM-F, and SD conceived and designed the experiments and analytical approaches; GM-F performed the animal trial; GM-F, JC, and CY analyzed biological samples. SD, MM, and GM-F analyzed the data. GM-F, CM, MM, and SD wrote the manuscript. All authors agree to be accountable for all aspects of the work.

## Conflict of Interest Statement

The authors declare that the research was conducted in the absence of any commercial or financial relationships that could be construed as a potential conflict of interest.
