# Supplementary material for: Methane Inhibition Alters the Microbial Community, Hydrogen Flow, and Fermentation Response in the Rumen of Cattle
Source: Front Microbiol. 2016 Jul 19;7:1122. doi: 10.3389/fmicb.2016.01122 (PMC4949212; doi:10.3389/fmicb.2016.01122)
Supplement: Supplementary file 1 [file Data_Sheet_1.DOCX]

***Supplementary Material***

**Methane inhibition alters the microbial community, hydrogen flow and fermentation response in the rumen of cattle**

**Gonzalo Martinez-Fernandez^1*^, Stuart E. Denman^1^, Chunlei Yang^2^, Jane Cheung^1^, Makoto Mitsumori^3^ and Christopher S. McSweeney^1^**

^1^ CSIRO, Agriculture and Food, Queensland Bioscience Precinct, St Lucia, QLD, Australia

^2^ Institute of Dairy Science, MoE Key Laboratory of Molecular Animal Nutrition, College of Animal Sciences, Zhejiang University, Hangzhou, China

^3^National Institute of Livestock and Grassland Science, Tsukuba, Ibaraki, Japan

*** Correspondence:** Gonzalo Martinez Fernandez, CSIRO, Agriculture and Food, Queensland Bioscience Precinct, 306 Carmody Road, St Lucia, QLD, 4067, Australia. Email:gonzalo.martinezfernandez@csiro.au

# Supplementary Figures and Tables

- 1. **Supplementary tables**

**Supplementary table 1.** CCD (mid dose) effects on rumen metabolites (picomoles/mL rumen fluid) on animals fed with hay:concentrate or hay diet 3 h after feeding.

|  | **Hay:Concentrate** | | | |  | **Hay** | | | |
| --- | --- | --- | --- | --- | --- | --- | --- | --- | --- |
| **Metabolites** | **Control** | **Mid dose** | **SEM** | ***P*-value** |  | **Control** | **Mid dose** | **SEM** | ***P*-value** |
| **Amino Acids & Amines** |  |  |  |  |  |  |  |  |  |
| Asparagine | 0.58 | 0.58 | 0.13 | *n.s.* |  | 0.22 | 0.43 | 0.07 | *** |
| Serine | 11 | 14 | 2.14 | *n.s.* |  | 5.4 | 7.6 | 0.79 | *** |
| Homoserine | 2.6 | 4.3 | 0.66 | *** |  | 1.0 | 3.0 | 0.61 | **** |
| Glycine | 13 | 20 | 3.9 | *n.s.* |  | 9.2 | 13.4 | 1.7 | **** |
| Alanine | 0.32 | 0.27 | 0.20 | *n.s.* |  | 0.63 | 0.04 | 0.23 | *t* |
| Aspartate | 26 | 56 | 11 | *** |  | 18 | 53 | 10 | **** |
| Glutamate | 54 | 78 | 9.2 | *t* |  | 53 | 61 | 6.2 | *n.s.* |
| Beta-Alanine | 3.1 | 4.3 | 1.1 | *n.s.* |  | 1.2 | 3.1 | 0.75 | *t* |
| Threonine | 8.7 | 12 | 1.6 | *n.s.* |  | 4.4 | 7.6 | 0.95 | **** |
| GABA | 3.0 | 3.3 | 0.65 | *n.s.* |  | 1.5 | 2.7 | 0.38 | **** |
| Proline | 13 | 21 | 3.0 | *** |  | 8.1 | 15.7 | 2.4 | **** |
| Lysine | 18 | 30 | 3.6 | *n.s.* |  | 9.8 | 15 | 1.8 | *** |
| Putrescine | 15 | 11 | 5.5 | *n.s.* |  | 3.5 | 2.6 | 0.36 | *t* |
| Tyrosine | 2.8 | 4.6 | 2.7 | *n.s.* |  | 1.5 | 2.6 | 0.32 | **** |
| Methionine | 3.9 | 5.5 | 0.80 | *n.s.* |  | 2.0 | 3.1 | 0.32 | ***** |
| Valine | 8.9 | 24 | 5.8 | *** |  | 4.2 | 11 | 2.0 | **** |
| Tyramine | 1.9 | 1.8 | 0.26 | *n.s.* |  | 0.2 | 1.4 | 0.38 | *** |
| Isoleucine | 5.1 | 12 | 2.9 | *** |  | 2.1 | 6.0 | 1.1 | ***** |
| Leucine | 5.5 | 12 | 2.8 | *t* |  | 2.5 | 5.6 | 0.88 | ***** |
| Phenylalanine | 3.2 | 6.2 | 1.6 | *n.s.* |  | 1.2 | 2.5 | 0.35 | ***** |
| Tryptophan | 0.97 | 1.1 | 0.08 | *n.s.* |  | 0.5 | 0.7 | 0.05 | *** |
| Phenethylamine | 0.78 | 0.87 | 0.26 | *n.s.* |  | 0.6 | 3.7 | 0.87 | ***** |
| Tryptamine | 0.35 | 0.40 | 0.08 | *n.s.* |  | 0.02 | 0.29 | 0.08 | **** |
| **Organic Acids** |  |  |  |  |  |  |  |  |  |
| Malonate | 19.0 | 22.7 | 3.07 | *n.s.* |  | 19.9 | 26.4 | 1.9 | **** |
| Nicotinic Acid | 9.1 | 11.3 | 1.7 | *n.s.* |  | 4.5 | 6.9 | 0.77 | *** |
| Fumarate_2TMS | 1.9 | 2.4 | 0.47 | *n.s.* |  | 1.45 | 2.50 | 0.29 | ***** |
| Pipecolate_2TMS | 0.8 | 3.2 | 0.99 | *t* |  | 0.92 | 2.53 | 0.55 | *** |
| Malate | 4.2 | 12.4 | 4.4 | *n.s.* |  | 3.8 | 6.2 | 0.70 | **** |
| Salicylate | 2.6 | 2.5 | 0.39 | *n.s.* |  | 3.1 | 4.5 | 0.46 | **** |
| **Sugars** |  |  |  |  |  |  |  |  |  |
| S1_Xylose | 2.5 | 2.5 | 0.18 | *n.s.* |  | 2.0 | 2.5 | 0.14 | ***** |
| Xylose_2 | 1.5 | 1.5 | 0.14 | *n.s.* |  | 1.2 | 1.7 | 0.15 | ***** |
| S3_Ribose | 3.5 | 4.8 | 0.40 | **** |  | 4.8 | 5.7 | 0.29 | *** |
| Xylitol | 0.5 | 0.6 | 0.05 | *n.s.* |  | 0.56 | 0.72 | 0.05 | **** |
| S2_arabitol | 2.1 | 4.5 | 0.66 | ***** |  | 4.6 | 6.6 | 0.66 | *** |
| Fucose MX1 | 1.0 | 0.9 | 0.08 | *n.s.* |  | 0.78 | 0.88 | 0.04 | *t* |
| Fructose 1 | 6.4 | 8.6 | 1.3 | *n.s.* |  | 1.45 | 2.28 | 0.30 | *** |
| S4_Galactose | 0.6 | 0.7 | 0.07 | *n.s.* |  | 0.49 | 0.64 | 0.04 | ***** |
| S3_Inositol | 0.46 | 1.13 | 0.20 | **** |  | 0.30 | 0.67 | 0.12 | *** |
| Uric acid | 2.33 | 1.95 | 0.16 | *t* |  | 2.09 | 1.90 | 0.08 | *n.s.* |
| Ferulic Acid | 2.8 | 2.8 | 0.29 | *n.s.* |  | 3.15 | 3.57 | 0.16 | *t* |
| S2_Glucose-6-P MX1 | 7.3 | 3.5 | 1.4 | **** |  | 3.50 | 3.85 | 0.20 | *n.s.* |
| S1_Sucrose | 0.63 | 0.70 | 0.12 | *n.s.* |  | 0.76 | 1.07 | 0.13 | *t* |

*** (P < 0.001); ** (P < 0.01); * (P < 0.05); *t* (P < 0.1); *n.s.* (P > 0.1)

**Supplementary table 2.** CCD (mid dose) effects on metabolites profile (fold change from control) on animals fed a hay:concentrate or hay diet 3 h after feeding.

|  | **Hay:Concentrate** | | |  | **Hay** | | |
| --- | --- | --- | --- | --- | --- | --- | --- |
|  | Control | Mid dose | SEM |  | Control | Mid dose | SEM |
| **Organic Acids** |  |  |  |  |  |  |  |
| Lactic acid | 1 | 0.27 | 0.31 |  | 1 | 1.2 | 0.10 |
|  |  |  |  |  |  |  |  |
| **Fatty Acids and sterols** |  |  |  |  |  |  |  |
| Hexanoic acid | 1 | 2.6^t^ | 0.21 |  | 1 | 2.5* | 0.19 |
| Heptanoic acid | 1 | 5.0^t^ | 0.30 |  | 1 | 4.1* | 0.27 |
| Heptadecanoate | 1 | 1.2 | 0.15 |  | 1 | 1.7* | 0.12 |
| Octadecanol | 1 | 2.2** | 0.10 |  | 1 | 2.6*** | 0.04 |
|  |  |  |  |  |  |  |  |
| **Nucleic acids** |  |  |  |  | 1 |  |  |
| Inosine | 1 | 4.4^t^ | 0.30 |  | 1 | 2.2* | 0.17 |
| Hypoxanthine | 1 | 21^t^ | 0.48 |  | 1 | 5.0*** | 0.25 |

*** (P < 0.001); ** (P < 0.01); * (P < 0.05); t (P < 0.1);

- 1. **Supplementary Figures**

**A)**

**
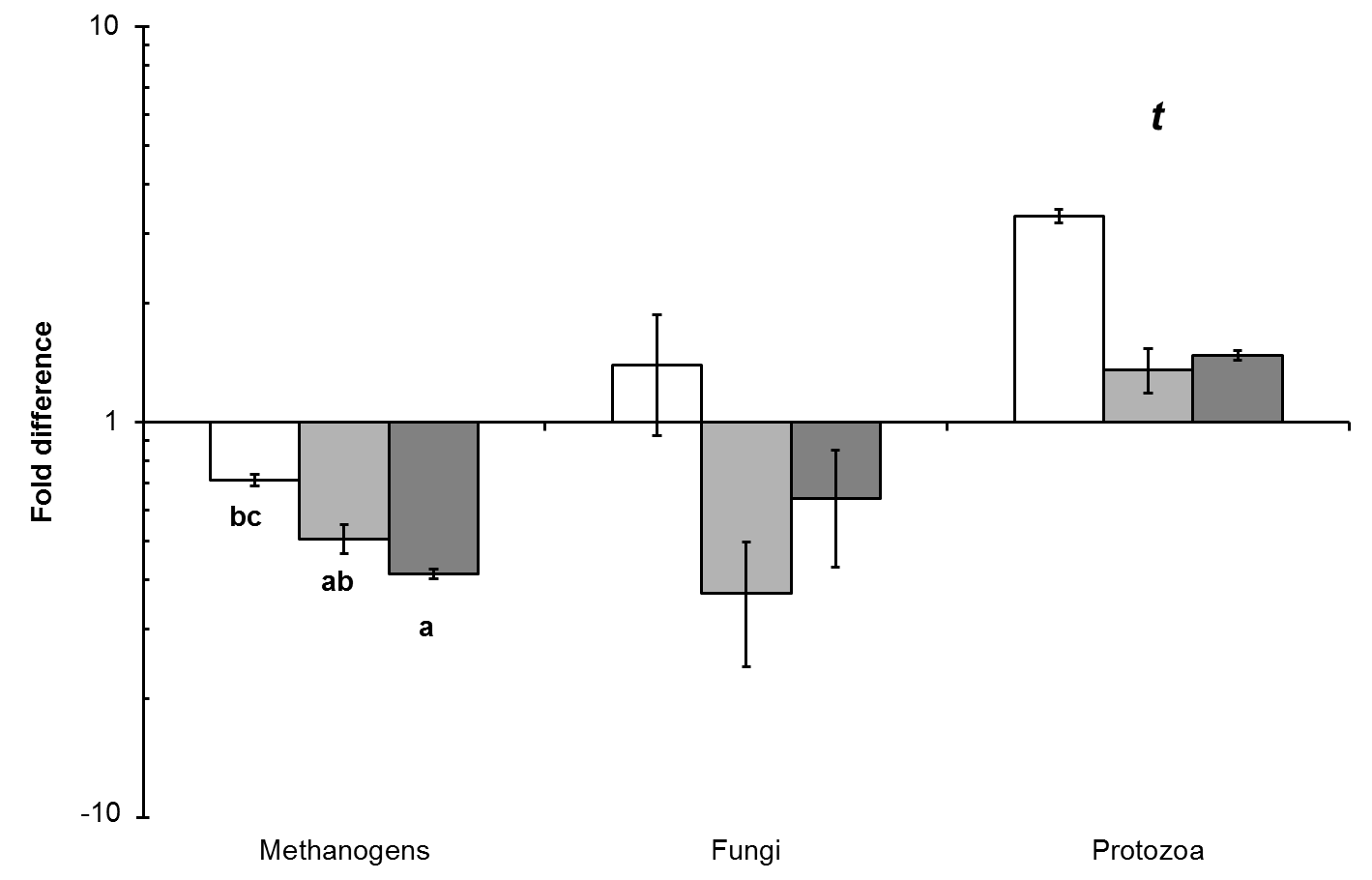
**
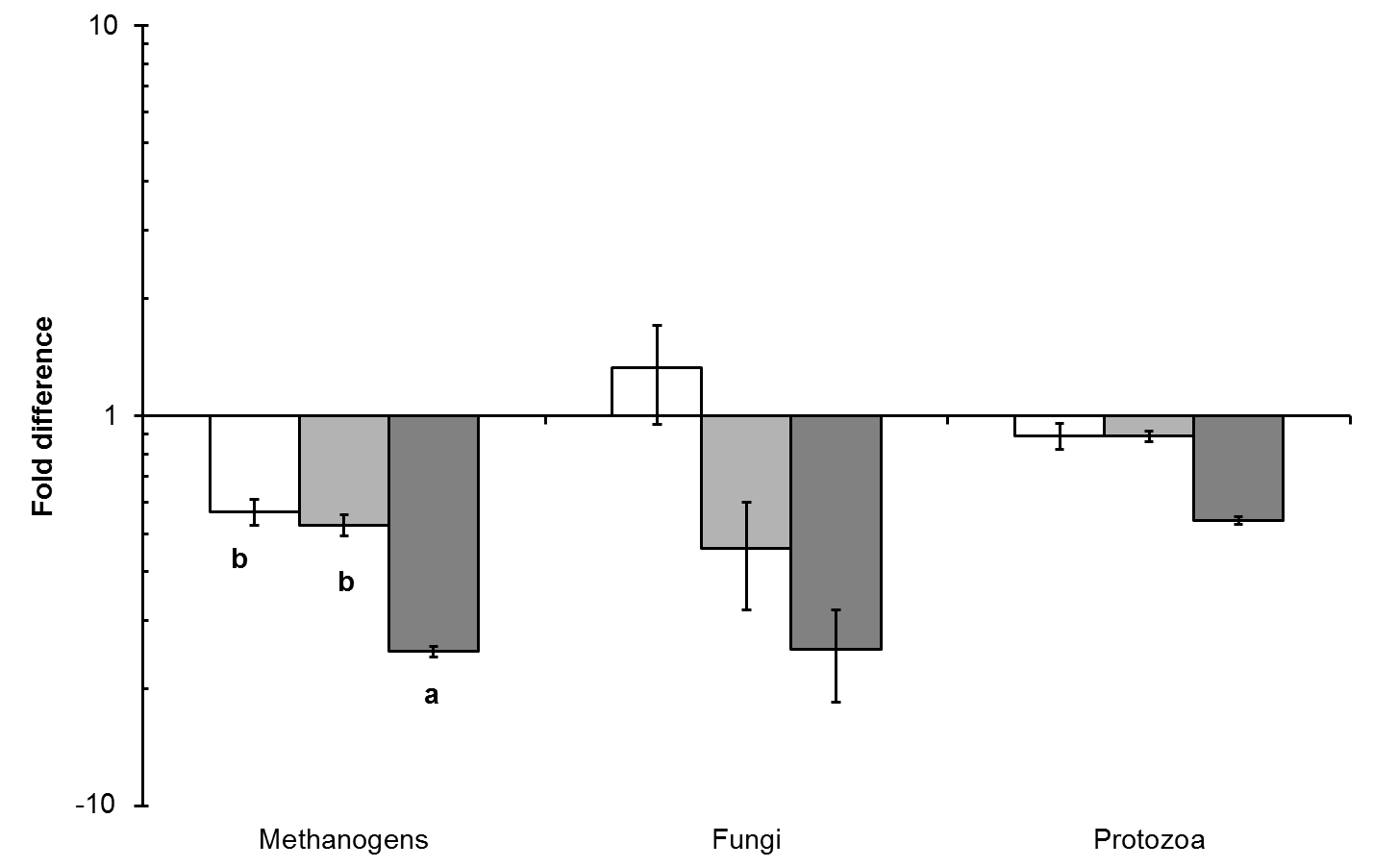


**B)**

**Supplemantary figure 1.** Quantitative PCR analysis of methanogens, protozoa and anaerobic fungi population changes in response to doses of CCD (low ; white, mid; grey and high; black) in animals fed a hay:concentrate diet a) or hay diet b). ^a,b^ Letters denote significant differences between treatments, bars that do not share the same letter for a species are significantly different from each other (P < 0.05). ^t^ Denote a trend between treatments (P < 0.1). The y-axis denotes fold change from control period.

**B)**

**A)**

##
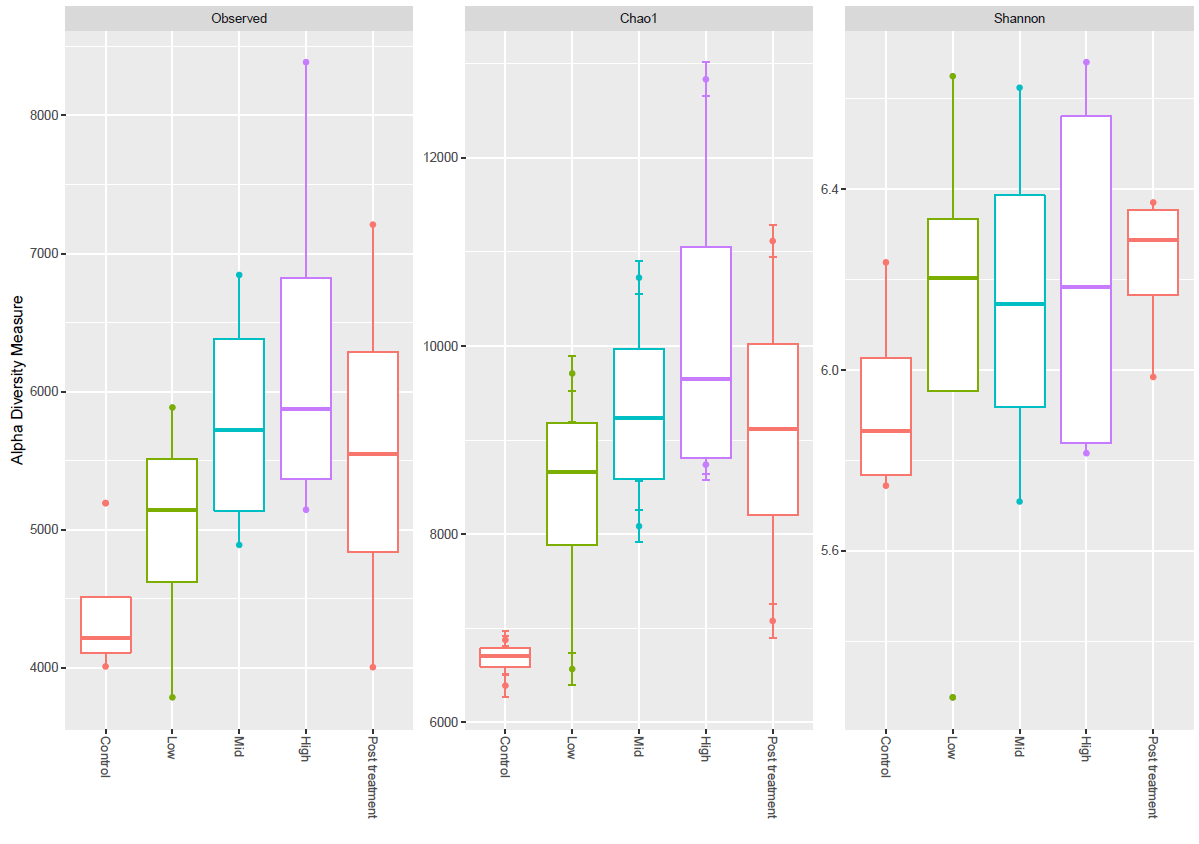

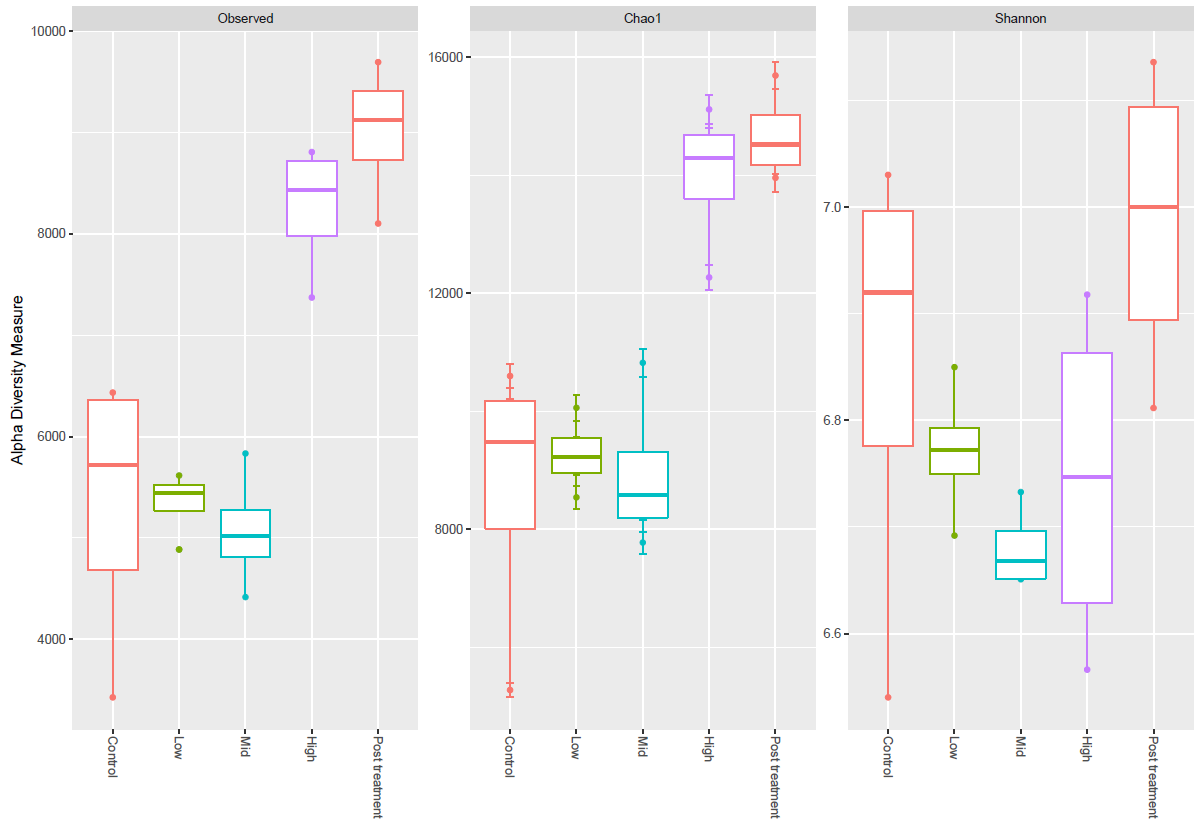


**Observed**

**Chao1**

**Shannon**

**Observed**

**Chao1**

**Shannon**

## Supplementary Figure 2 Alpha diversity measures for rumen microbiomes fed with hay:concentrate (A) or hay (B) diets at control, post-treatment, low, mid, and high doses of CCD illustrating the total observed taxonomic units (Observed), the Chao1estimates (Chao1) and the Shannon diversity index (Shannon). Boxplots indicate variance within the sampled animals with the box boundaries showing the first and third quartiles, the median value indicated as a horizontal line and the whiskers extend to 1.5 times the interquartile range.

**A)**

**B)**


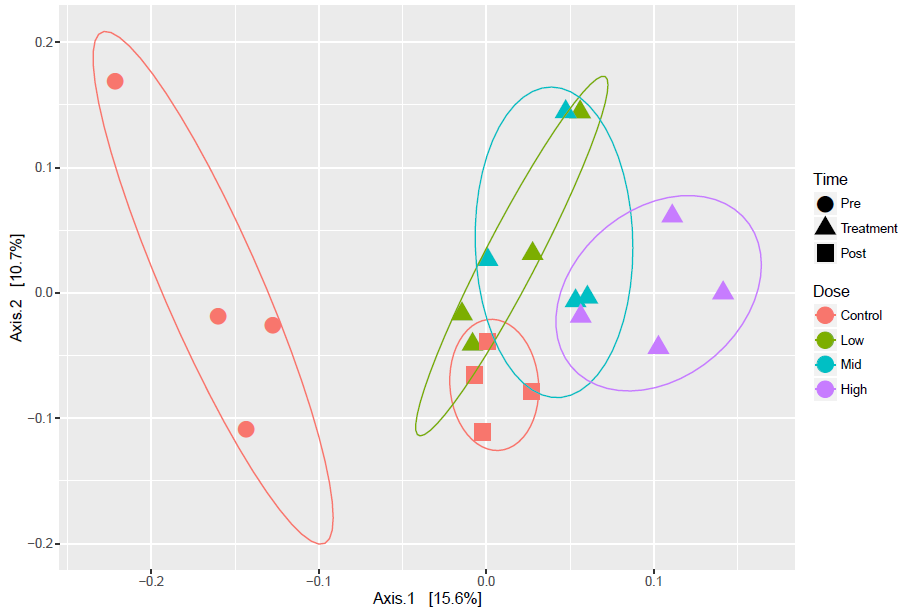

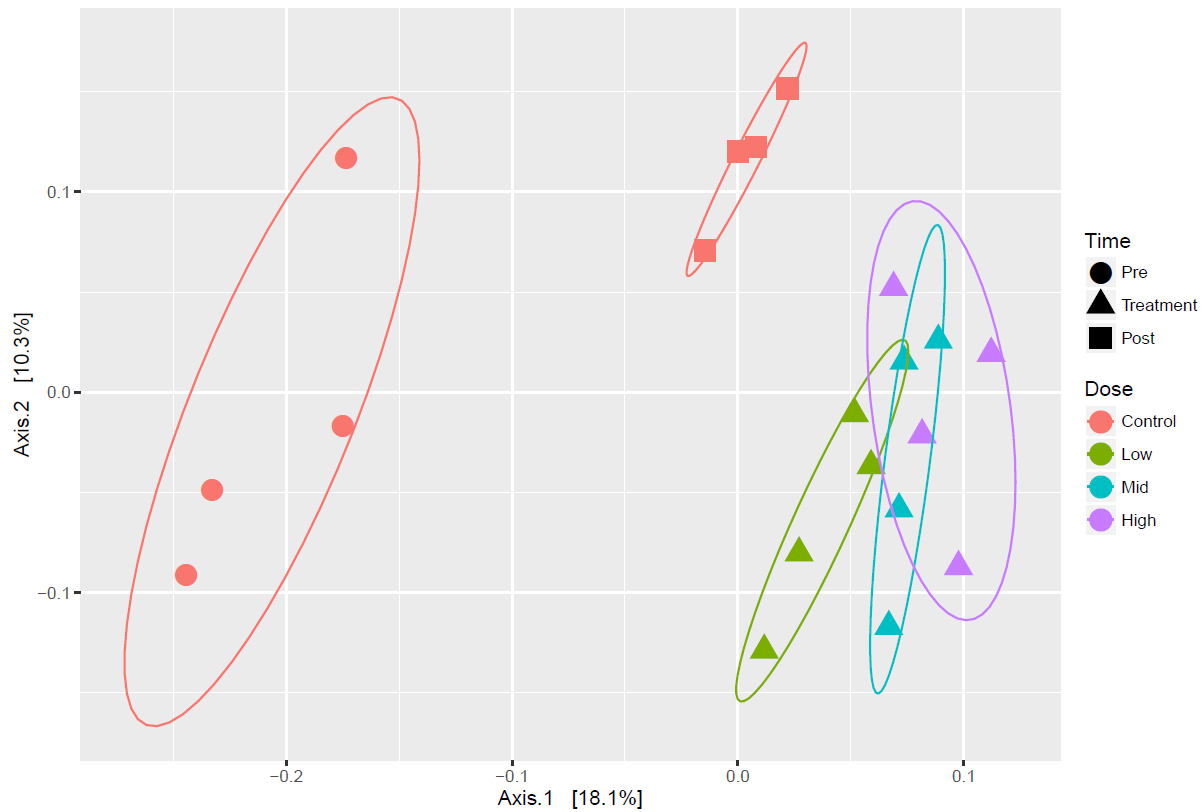


**Supplementary figure 3**. Principle Coordinate Analysis comparing changes in microbial OTU classification based on unweighted Unifrac calculations for control (●), post-treatment (■) and CCD (▲) treatments at low (Green), medium (blue) and high (purpure) doses of CCD in animals fed with hay:concentrate (A) or hay (B) diets.

##
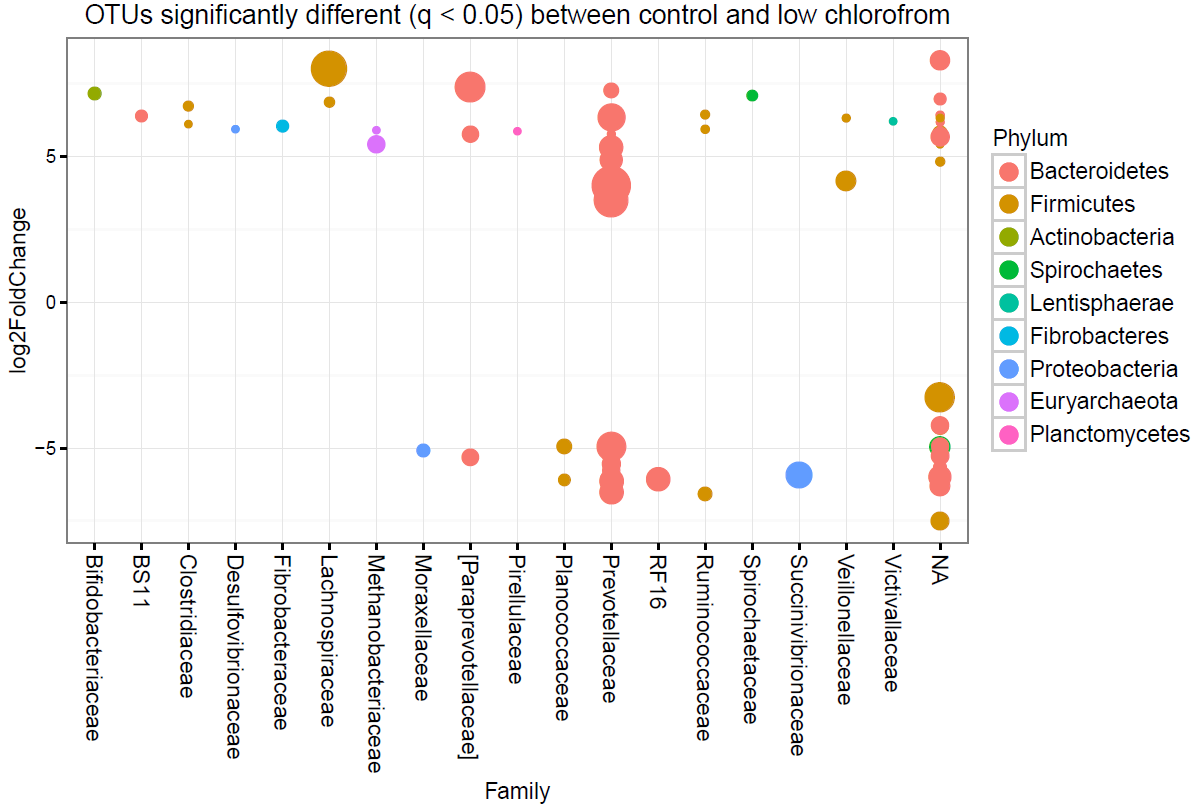


Log2 Fold change

**Supplementary Figure 4.** OTUs significantly different (q > 0.05 FDR) between control and CCD low dose from animals fed a hay:concetrate diet. Upper axis represents OTU’s with a log2 fold positive change for control relative to CCD treatment while the lower y axis is the negative fold change of the control relative to CCD low dose. Each point represents a single OTU colored by phylum and grouped on the x axis by taxonomic family level, size of point reflects the log2 mean abundance of the sequence data.


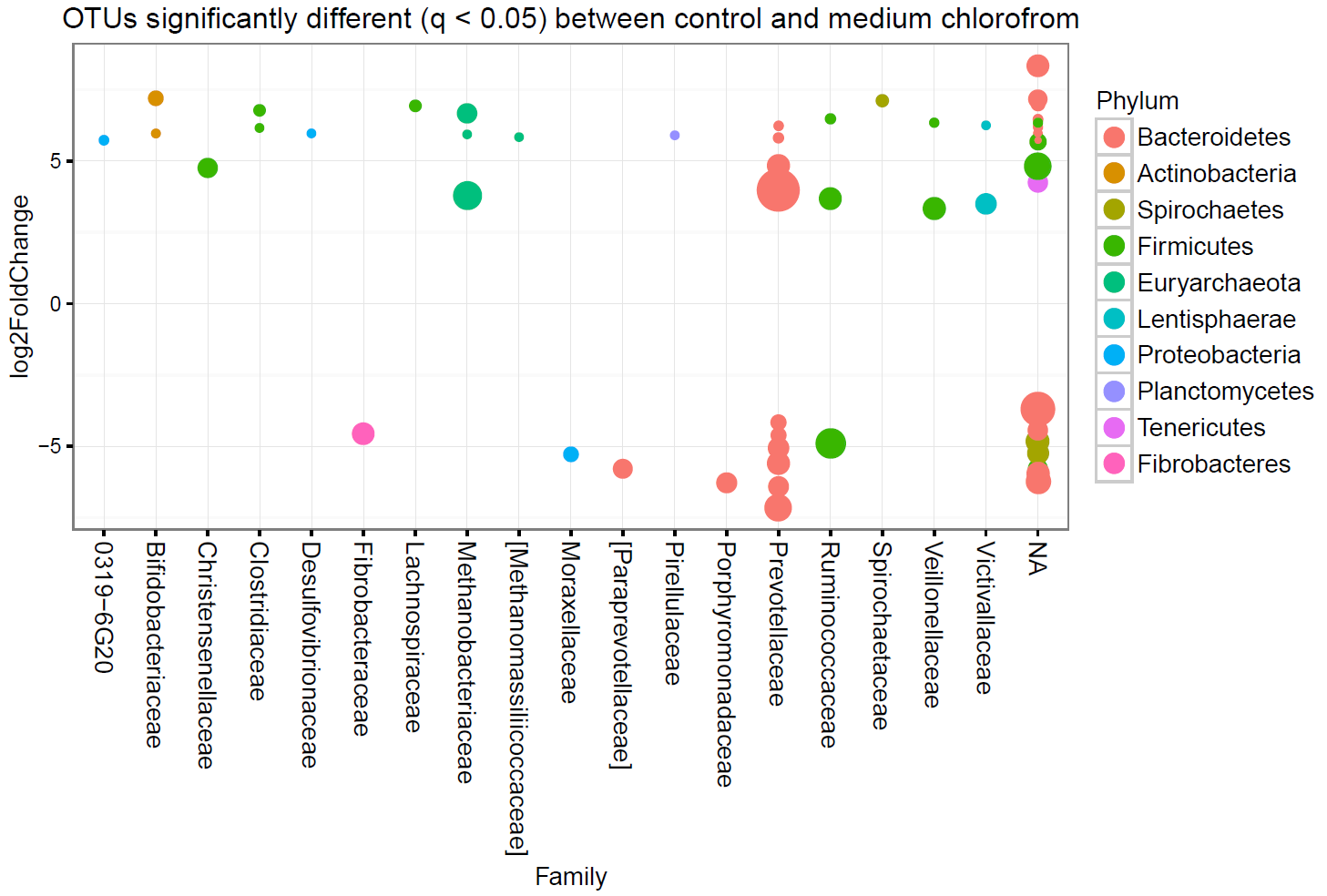


Log2 Fold change

**Supplementary Figure 5.** OTUs significantly different (q > 0.05 FDR) between control and CCD mid dose from animals fed a hay:concetrate diet. Upper axis represents OTU’s with a log2 fold positive change for control relative to CCD treatment while the lower y axis is the negative fold change of the control relative to CCD low dose. Each point represents a single OTU colored by phylum and grouped on the x axis by taxonomic family level, size of point reflects the log2 mean abundance of the sequence data.


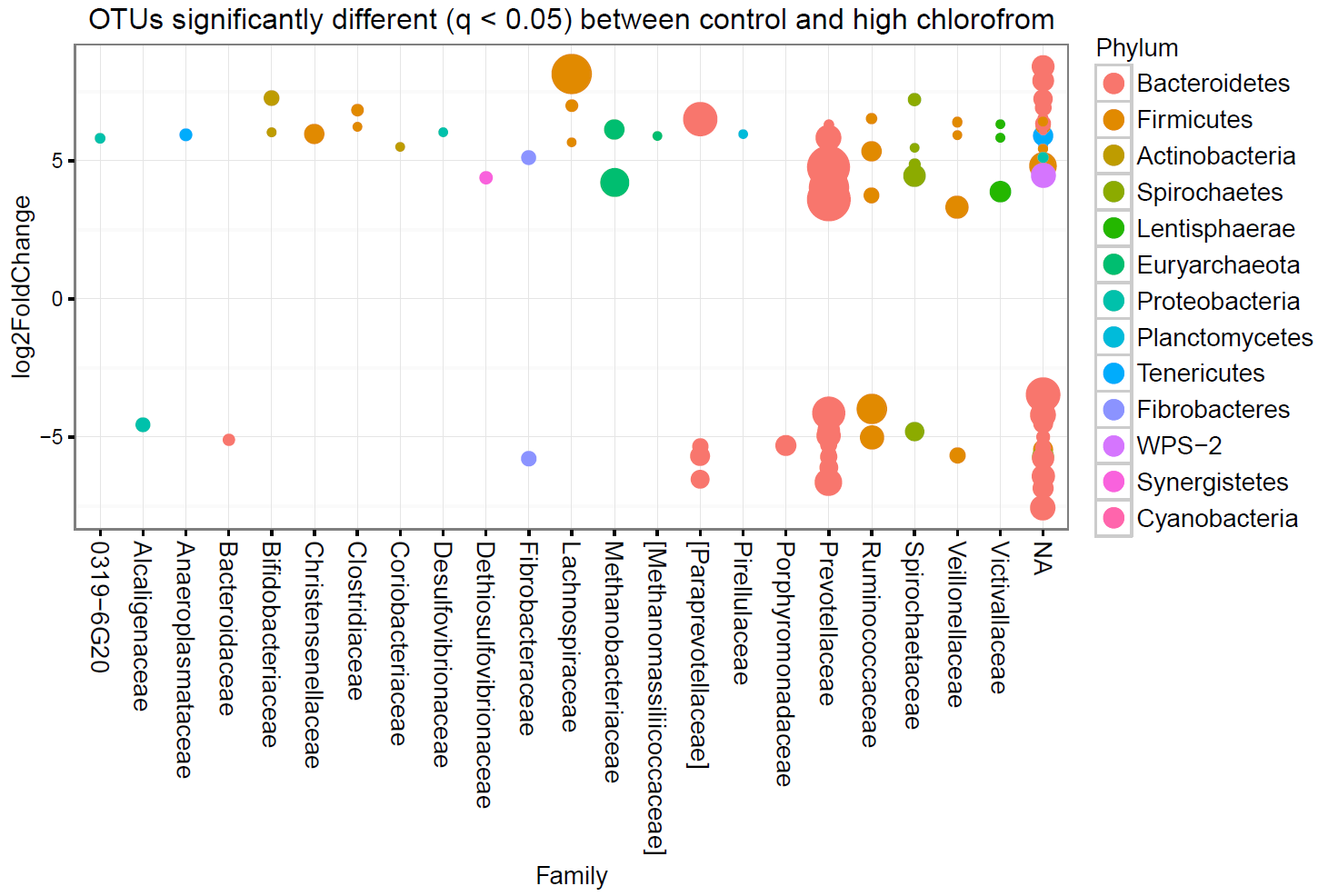


Log2 Fold change

**Supplementary Figure 6.** OTUs significantly different (q > 0.05 FDR) between control and CCD high dose from animals fed a hay:concetrate diet. Upper axis represents OTU’s with a log2 fold positive change for control relative to CCD treatment while the lower y axis is the negative fold change of the control relative to CCD low dose. Each point represents a single OTU colored by phylum and grouped on the x axis by taxonomic family level, size of point reflects the log2 mean abundance of the sequence data.


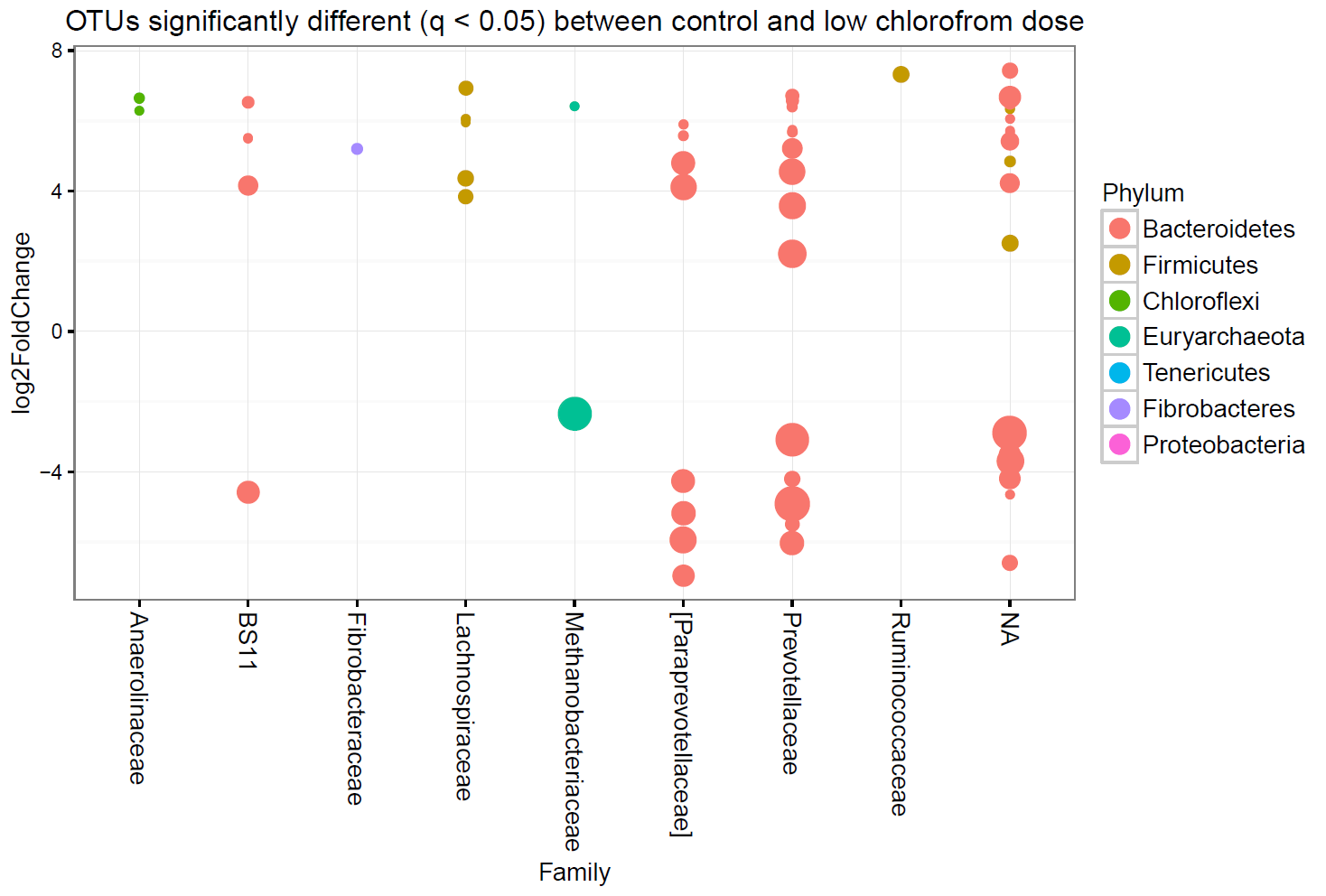


Log2 Fold change

**Supplementary Figure 7.** OTUs significantly different (q > 0.05 FDR) between control and CCD low dose from animals fed a hay diet. Upper axis represents OTU’s with a log2 fold positive change for control relative to CCD treatment while the lower y axis is the negative fold change of the control relative to CCD low dose. Each point represents a single OTU colored by phylum and grouped on the x axis by taxonomic family level, size of point reflects the log2 mean abundance of the sequence data.


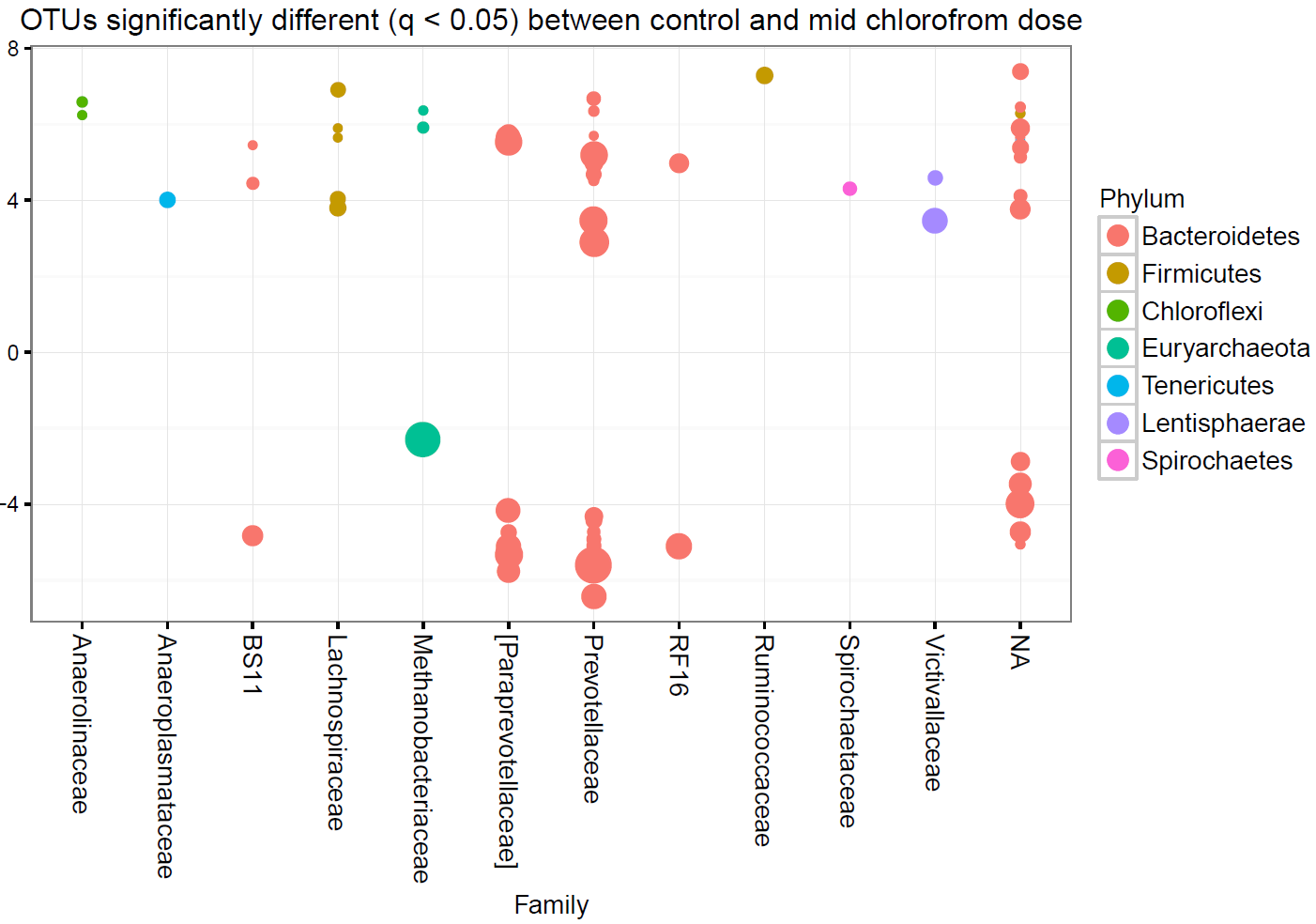


Log2 Fold change

**Supplementary Figure 8.** OTUs significantly different (q > 0.05 FDR) between control and CCD mid dose from animals fed a hay diet. Upper axis represents OTU’s with a log2 fold positive change for control relative to CCD treatment while the lower y axis is the negative fold change of the control relative to CCD low dose. Each point represents a single OTU colored by phylum and grouped on the x axis by taxonomic family level, size of point reflects the log2 mean abundance of the sequence data.


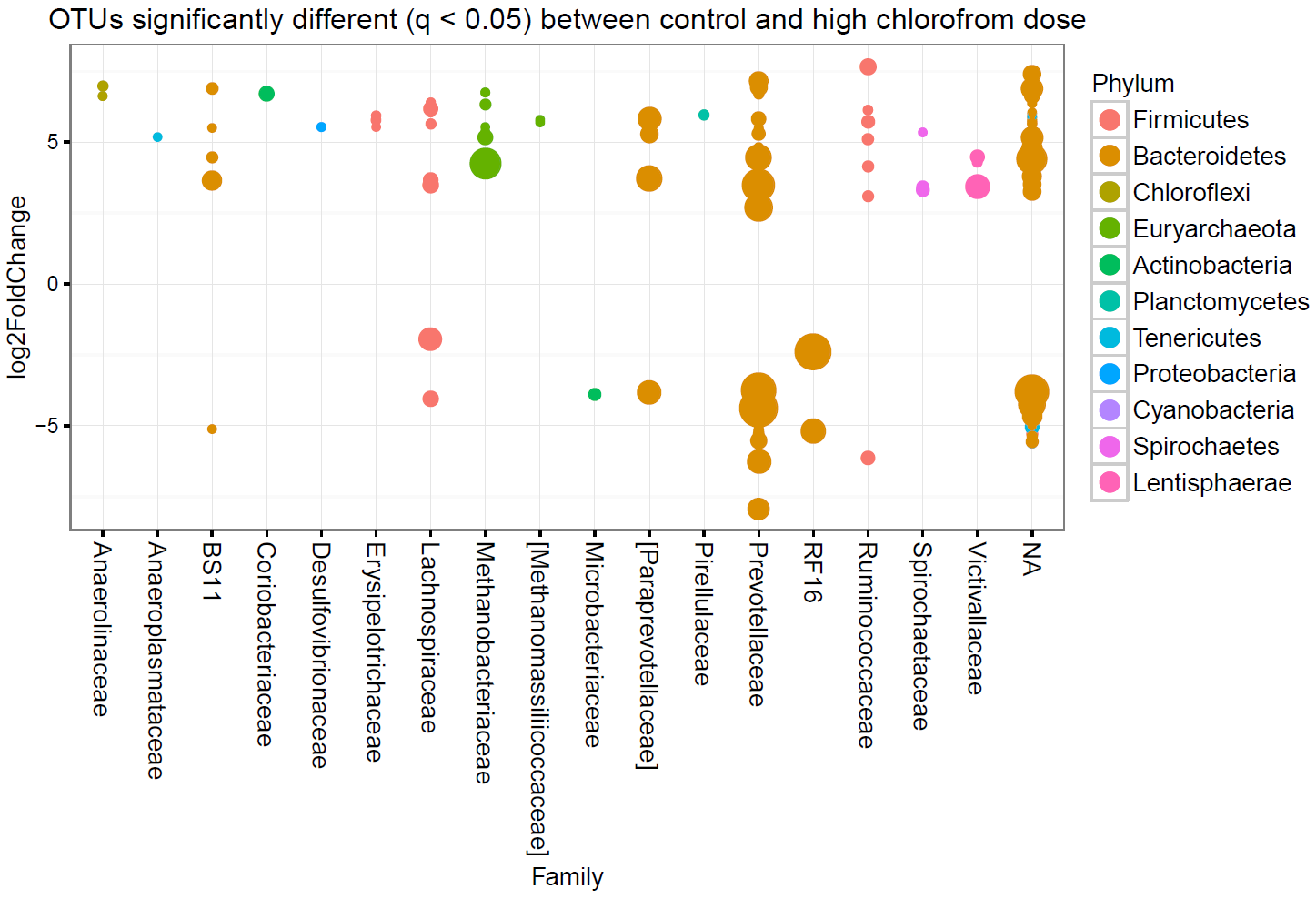


Log2 Fold change

**Supplementary Figure 9.** OTUs significantly different (q > 0.05 FDR) between control and CCD high dose from animals fed a hay diet. Upper axis represents OTU’s with a log2 fold positive change for control relative to CCD treatment while the lower y axis is the negative fold change of the control relative to CCD low dose. Each point represents a single OTU colored by phylum and grouped on the x axis by taxonomic family level, size of point reflects the log2 mean abundance of the sequence data.
